# Molecular Dynamics of "Fuzzy" Transcriptional Activator-Coactivator Interactions

**DOI:** 10.1371/journal.pcbi.1004935

**Published:** 2016-05-13

**Authors:** Natalie S. Scholes, Robert O. J. Weinzierl

**Affiliations:** Imperial College London, Department of Life Sciences, London, United Kingdom; Ottawa University, CANADA

## Abstract

Transcriptional activation domains (ADs) are generally thought to be intrinsically unstructured, but capable of adopting limited secondary structure upon interaction with a coactivator surface. The indeterminate nature of this interface made it hitherto difficult to study structure/function relationships of such contacts. Here we used atomistic accelerated molecular dynamics (aMD) simulations to study the conformational changes of the GCN4 AD and variants thereof, either free in solution, or bound to the GAL11 coactivator surface. We show that the AD-coactivator interactions are highly dynamic while obeying distinct rules. The data provide insights into the constant and variable aspects of orientation of ADs relative to the coactivator, changes in secondary structure and energetic contributions stabilizing the various conformers at different time points. We also demonstrate that a prediction of α-helical propensity correlates directly with the experimentally measured transactivation potential of a large set of mutagenized ADs. The link between α-helical propensity and the stimulatory activity of ADs has fundamental practical and theoretical implications concerning the recruitment of ADs to coactivators.

## Author Summary

The regulated transcription of eukaryotic genes is governed by gene-specific transcription factors that contain activation domains to stimulate the expression of nearby genes. Activation domains are unable to take up a defined three-dimensional conformation. Nevertheless, as we demonstrate in our study, molecular dynamics simulations reveal that the key docking point of such domains (centered around several large hydrophobic amino acid sidechains) folds into fluctuating α-helical conformations. Analysis of published data shows that this tendency of adopting such local structures correlates directly with stimulation activity. We also investigate the interaction of these structurally unstable domains with a coactivator interaction partner. Computational simulations are ideally suited for analysing the rapidly changing, "fuzzy" interactions occurring between these protein partners. We gained new insights into the competitive nature of the key hydrophobic sidechains in binding to a pocket on the coactivator surface and documented for the first time the rapidly changing movements of an activation domain during these interactions.

## Introduction

**Fig 1:**
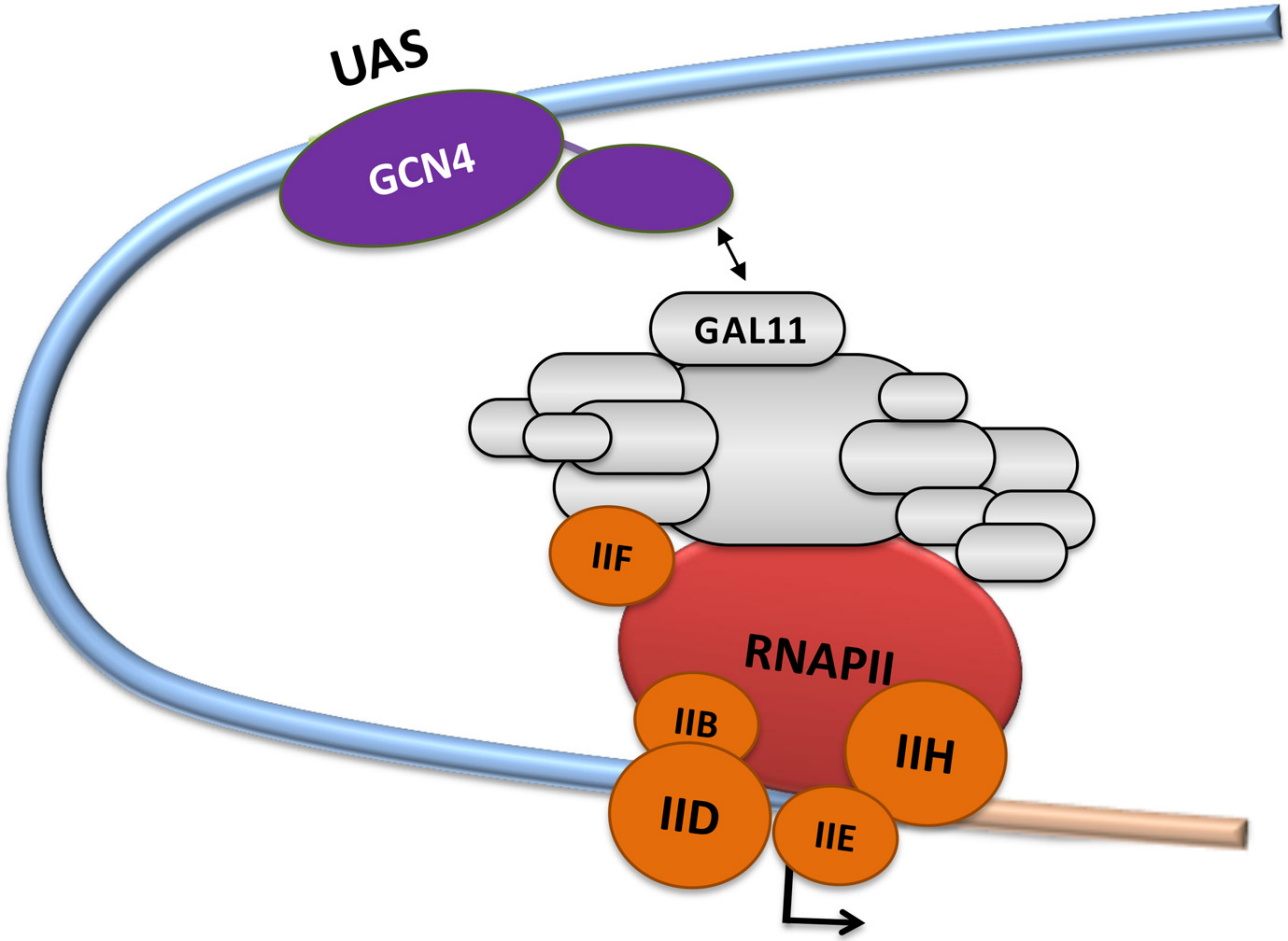
Transcriptional activation via the Mediator complex. The schematic diagram shows the gene-specific transcription factor GCN4 bound sequence-specifically to a target site ("Upstream Activation Site"; "UAS"). The basal transcriptional machinery (including RNA polymerase II [RNAPII]; basal transcription factors TFIIB, TFIID, TFIIE, TFIIF and TFIIH; the multi-subunit "Mediator Complex") assembles around the transcription start site indicated by an arrow. The GAL11 subunit of the Mediator is specifically identified. The regulatory protein-protein interaction of the GCN4-activation domain (cAD) with GAL11 is represented by a two-headed arrow.

Control of gene expression plays a crucial role throughout all three evolutionary domains of life, allowing cells to establish cellular identity, adapt to environmental challenges and prevent diseases caused by misregulation of transcription [1]. The expression of the genome is controlled predominantly by a network of gene-specific transcription factors (GSTFs) that, after binding to target sites on DNA, regulate the rate of expression of nearby genes. GSTFs performing as transcriptional activators usually contain one or multiple activation domains (ADs; [2]) that orchestrate localized remodelling of the chromatin structure, enhanced recruitment of components of the basal transcriptional machinery on the core promoter and/or stimulate promoter escape and subsequent elongation events [3–6]. These activities typically require binding of the ADs to coactivators that integrate and convey activation signals to other components of the transcriptional machinery [6,7]. The Mediator complex surrounding the basal transcriptional machinery during transcription initiation [8–11] contains coactivators that have been shown experimentally to interact with ADs to regulate gene-specific transcription (Fig 1; [11–13]).

**Fig 2:**
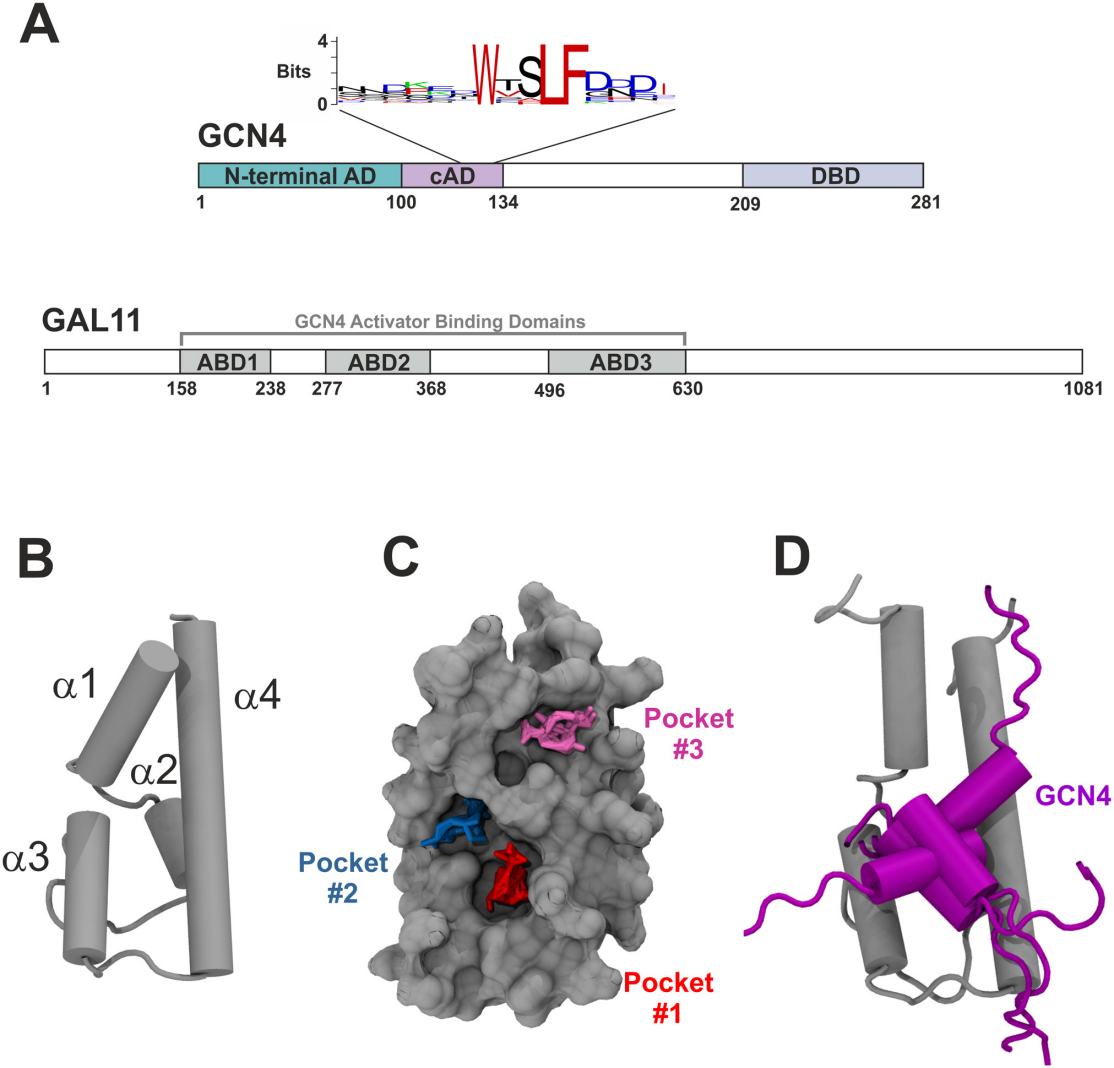
Transcriptional activation of GCN4 via Mediator GAL11. **A.** Domain organization of GCN4 (top) and GAL11 (bottom). The positions of the two tandem activation domains (N-terminal AD, central AD [cAD]) and the DNA-binding domain (DBD) of GCN4 are illustrated. A sequence logo based on GCN4 orthologs from 29 different species (see S1 Text for sequence alignments) shows the absolute conservation of three large hydrophobic residues (corresponding to W120, L123 and F124 in *Saccharomyces cerevisiae* GCN4). GAL11 contains three domains,ABD1, ABD2 and ABD3, that each can bind GCN4 independently. **B.** Structure of GAL11-ABD1. Cartoon presentation of the uncomplexed structure showing positions of the four separate α-helices (α1 to α4 from N- to C-terminus). **C.** Surface view showing the location of three deep pockets that display a capacity for binding hydrophobic side chains of partner proteins. Liquorice representations of probe clusters of small organic compounds are shown fitted into three distinct pockets (red = "Pocket#1"; blue = "Pocket#2"; magenta = "Pocket#3") mapped by a computational solvent mapping program [32]. The model shows GAL11-ABD1 residues 164–233. **D.** Model of the GCN4-cAD bound to GAL11-ABD1. The GAL11-ABD1 and GCN4 structures are shown in silver and purple, respectively. Five different positions, reflecting orientations at 200 ns intervals of aMD_no1, are drawn for the GCN4 cAD in cartoon representation.

While more than 50 common structural motifs have been described for the DNA-binding domains, the available knowledge concerning the structure and function of ADs is comparatively limited [14]. The first ADs described almost three decades ago were shown to be both necessary and sufficient to confer the transcriptional stimulatory properties [2,15]. From a structural perspective, ADs are often characterized by their unusual primary amino acid sequence abundant in acidic amino acids, glutamine or proline residues [14–17]. The enrichment for such amino acids is thought to discourage the formation of higher order structures and thus results in an intrinsically disordered structure ("acid blobs and negative noodles" or "polypeptide lasso" structures [18–20]). In turn, the intrinsic disorder allows ADs to interact in a highly adaptable manner with a range of coactivators, culminating in a synergistic regulation of the basal transcriptional machinery by one or multiple activators (Fig 1; [21,22]). The affinity of AD-coactivator binding is reasonably high (low micro- to high nanomolar range [12,21,23]) and results in interactions lasting for several milliseconds. NMR-studies provided structural insights into a various aspects of AD-coactivator complexes (TFIID/Taf40-VP16 [24]; TFIIH/Tfb1-VP16 (PDB#2K2U [23]); NcoA1-STAT6 (PDB#1OJ5 [25]); MDM2-p53 (PDB#1YCQ [26]); CBP-CREB (PDB#1KDX [27]; MED25/VP16 (PDB#2XNF [12] and 2KY6 [13]; GAL11-GCN4 (PDB#2LPB [11]). Site-directed mutagenesis and structural studies have shown that evolutionarily highly conserved bulky hydrophobic residues within ADs play a key structural role in mediating interactions with coactivators (Fig 2A and S1 Text, [16,23,24,26,28]). When bound to coactivators, ADs form a "fuzzy" family of stochastically related structures (Fig 2D, [29–31]).

Many of the yet unanswered questions regarding AD-coactivator interactions are challenging to address experimentally, especially those concerning the dynamic range of AD conformations over time, key interaction points on coactivator surfaces, the energetics of such interactions and the structures of ADs prior to binding coactivators. Computational approaches are highly effective to model such systems on the atomic level, to study their behavior and gain new mechanistic insights that consolidate present knowledge and guide future experimental work. Here we describe the results obtained from a series of long, fully atomistic molecular dynamics simulations focusing on the experimentally well-characterized GCN4-GAL11 system from *Saccharomyces cerevisiae*. Accelerated molecular dynamics (aMD) methods [33,34] provide powerful tools for investigating the binding of the ADs to their coactivator targets, as well as for studying the structural properties of ADs in isolation. We describe the structural interplay of AD-coactivator complexes and explore an extensive experimental data set based on synthetic AD variants to demonstrate a high degree of correlation between the α-helix propensity, degree of "fuzziness" and the transactivation potential.

## Results

### Microsecond simulations of the GAL11-GCN4 complex: Structural and energetic aspects

The yeast transcriptional activator GCN4 contains two tandemly arranged ADs (Fig 2A) that stimulate the expression of more than 70 "downstream" genes. The GCN4 ADs achieve this task by targeting a variety of components of the basal transcriptional machinery, including the coactivator GAL11 (also known as MED15) within the mediator complex [21]). GAL11 contains three structurally independent AD-binding domains ("Activator-Binding Domains" ["ABDs"]; Fig 2A). For one of these, ABD-1, a high-resolution structure shows a stable α-helical structure that includes a groove for interactions with ADs (PDB#2LPB; Fig 2B–2D; [11]). NOE and spin-labeling data of GAL11/ABD-1 complexed with the central AD of GCN4 (GCN4-cAD) were used to create several models illustrating the diversity of interaction between this coactivator and the AD. The bound cAD models contain a short helical stretch (encompassing GCN4 residues 116 to 124) that includes three large hydrophobic residues (W120, L123 and F124) highly conserved during evolution (Fig 2A and S1 Text). The coactivator GAL11/ABD-1 interaction surface displays three computationally detectable "hot spots" ("Pocket #1", "Pocket #2" and "Pocket #3") [32] that are distinguished by their concave topology and potentially become occupied by these particular GCN4 hydrophobic residues (Fig 2C).

We subjected PDB#2LPB-model 1 to extensive aMD simulations to gain deeper insight into various structural aspects, such as variation in AD secondary structure, orientation relative to the coactivator surface and energetic changes underpinning the conformational changes. Simulations were carried out as four independent replica runs with different initial Boltzmann distributions of particle velocities. Each simulation lasted for one microsecond, but the results reflect a period around two or three orders of magnitude longer due to the acceleration protocol used (that is, hundreds of microsecond- to millisecond-range; Table 1).

**Table 1:** Summary of MD simulations.

Simulated Structure	Simulation Name	Duration	#atoms
GAL11-ABD1/GCN4-cAD	GAL11-ABD1/GCN4-cAD _aMD_no1	1 μs	45,489
(PDB#2LPB-model 1)	GAL11-ABD1/GCN4-cAD _aMD_no2	1 μs	45,489
	GAL11-ABD1/GCN4-cAD _aMD_no3	1 μs	45,489
	GAL11-ABD1/GCN4-cAD _aMD_no4	1 μs	45,489
GCN4-cAD	GCN4_aMD_no1	1 μs	32,551
(*de novo* starting structure)	GCN4_aMD_no2	1 μs	32,551
	GCN4_aMD_no3	1 μs	32,551
	GCN4_aMD_no4	1 μs	32,551
GAL11-ABD1	GAL11-ABD1 _aMD_no1	1 μs	42,355
(PDB#2LPB; without GCN4)	GAL11-ABD1 _aMD_no2	1 μs	42,355
	GAL11-ABD1 _aMD_no3	1 μs	42,355
	GAL11-ABD1 _aMD_no4	1 μs	42,355
cAD-like07	cAD-like07_aMD_no1	1 μs	28,131
(*de novo* starting structure)			
cAD-like96	cAD-like96_aMD_no1	1 μs	28,424
(*de novo* starting structure)			
GAL11-ABD1/GCN4-cADlike96	GAL11-ABD1/cAD-like96_aMD_no1	1 μs	46,862
(PDB#2LPB; *in silico*	GAL11-ABD1/cAD-like96_aMD_no2	1 μs	46,862
mutagenized GCN4)	GAL11-ABD1/cAD-like96_aMD_no3	1 μs	46,862
	GAL11-ABD1/cAD-like96_aMD_no4	1 μs	46,862

**Fig 3:**
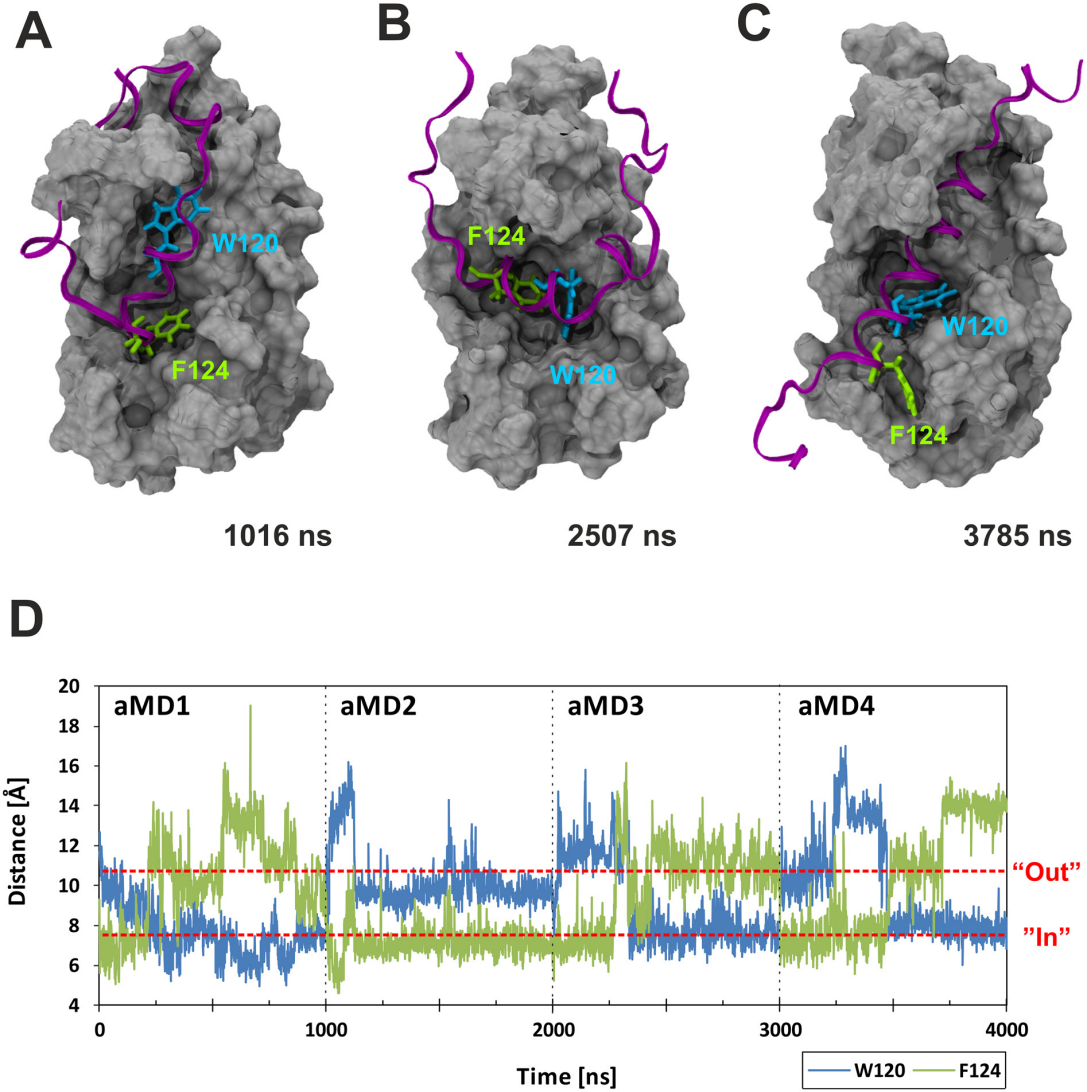
Differential GAL11-ABD1 pocket occupancy by GCN4-W120 and F124. Panels A—C show the GAL11-ABD1 in grey surface representation and the GCN4-cAD as a purple ribbon. The key hydrophobic residues GCN4-W120 and F124 are shown as liquorice models in light blue and green, respectively. The time-frame shown in the right corner of each panel corresponds to the 4 x 1000 ns aggregate trajectory shown in Panel D. **A.** GCN4-F124 located in the ABD1 pocket near the beginning of aMD_no2. **B.** Co-occupancy of GCN4-W120 and F124 inside the ABD1 pocket. Note that the cAD α-helix is additionally rotated by 90° in comparison to (A) and (C). **C.** GCN4-W120 located within the ABD1 pocket towards the end of aMD_no4. Note that this simulation, just like the other three started out with GCN4-F124 in the pocket. **D.** Distance measurement [Å] between residue GAL11-A216, which forms the floor of the ABD1 pocket, and GCN4-W120 (blue trace) or F124 (green trace). For this analysis the four aMD simulations trajectories were concatenated to a single trajectory before processing to allow cross-comparison of the data under identical conditions.

**Fig 4:**
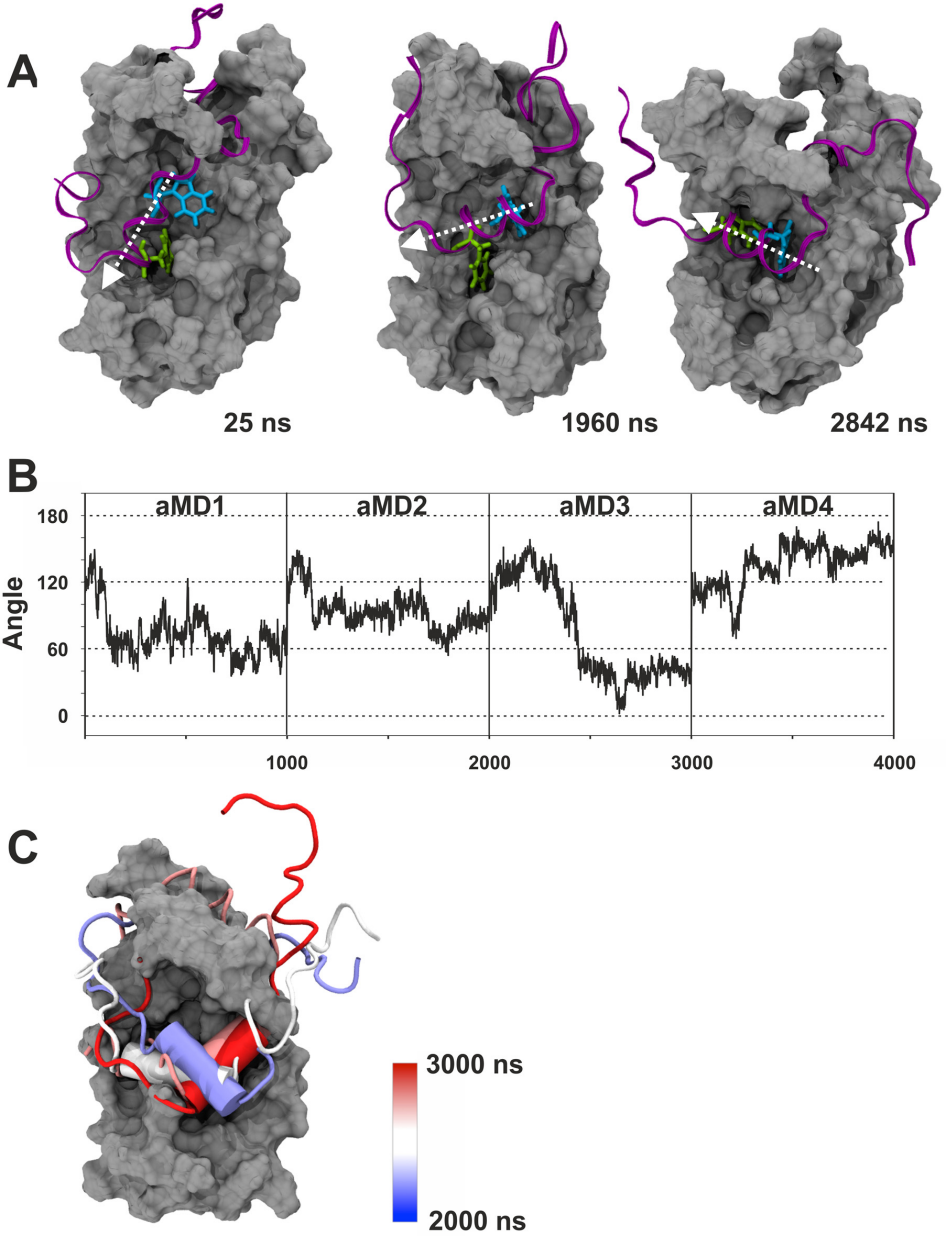
Changes in helical orientation of the GCN4-cAD relative to GAL11-ABD1. **A.** The three panels show the GAL11-ABD1 in silver surface representation and the GCN4-cAD as a purple ribbon. The key hydrophobic residues GCN4-W120 and F124 are shown as liquorice models in light blue and green, respectively. The time-frame shown in the right corner of each panel corresponds to the 4 x 1000 ns aggregate trajectory shown in Panel D. All representations are aligned to the long α-helix 4 of ABD1 (see Fig 2B for annotation of helices; residues GAL11-Q211 and L232 were chosen as vector endpoints for α-helix 4). The helical axis of GCN4 is marked by a white arrow. **B.** Measurements of the angle of α-helix 4 of ABD1 in relation to the GCN4-cAD α-helix during simulations GAL11-ABD1/GCN4-cAD _aMD_no1, no 2, no3 and no4. For the cAD α-helix, residues GCN4-S117 and F124 were selected. **C.** Time-dependent representation of the 90° rotation performed by cAD (cartoon representation, red to blue) in GAL11-ABD1/GCN4-cAD _aMD_no3. The different helical orientations (shown in cartoon representation) at different time points throughout the one microsecond simulation are superimposed on each other. The color of the helix indicates the time point according to the color gradient scale shown on the right. The GCN4 helix undergoes a number of different orientations (~100°, ~150°, ~50° and ~0° according to the criteria defined in B).

We were initially curious to see whether the aMD simulations would recreate the different binding states previously proposed by Brzovic *et al*. [11]. We used distance measurements between GCN4-W120 or GCN4-F124 relative to GAL11/ABD1-A126 (which forms the floor of Pocket #1; Fig 2B) to monitor pocket occupancy. The measurements show that the two key hydrophobic residues, in full agreement with the NMR-based models [11], behave in a switch-like manner and bind to GAL11/ABD1 in the three major binding states via a series of intermediate conformations (Fig 3). At various stages, the GAL11-ABD1 pocket is occupied by the sidechains of either GCN-4/W120 (Fig 3A) or F124 (Fig 3C), respectively. On several occasions, we observe a double occupancy (Fig 3B). A molecular movie illustrates a full time course of the dynamic change, including the changeover between W120 and F124 (S1 Movie). In addition to pocket occupancy state, the NMR-based models also postulate that the GCN4-cAD helical portion takes up several different orientations relative to GAL11-ABD1. Angular measurements of vectors characterizing the GCN4-cAD helix relative to GAL11-ABD1 α-helix 4 (Fig 4) correspond to orientations directly comparable to the previously described ones, but also suggest the presence of additional states representing transitional conformations. Because W120 and F124 act as pivot points in a comparable manner, the various pocket occupancy states and helix orientations observed do not appear to show any significant correlation.

**Fig 5:**
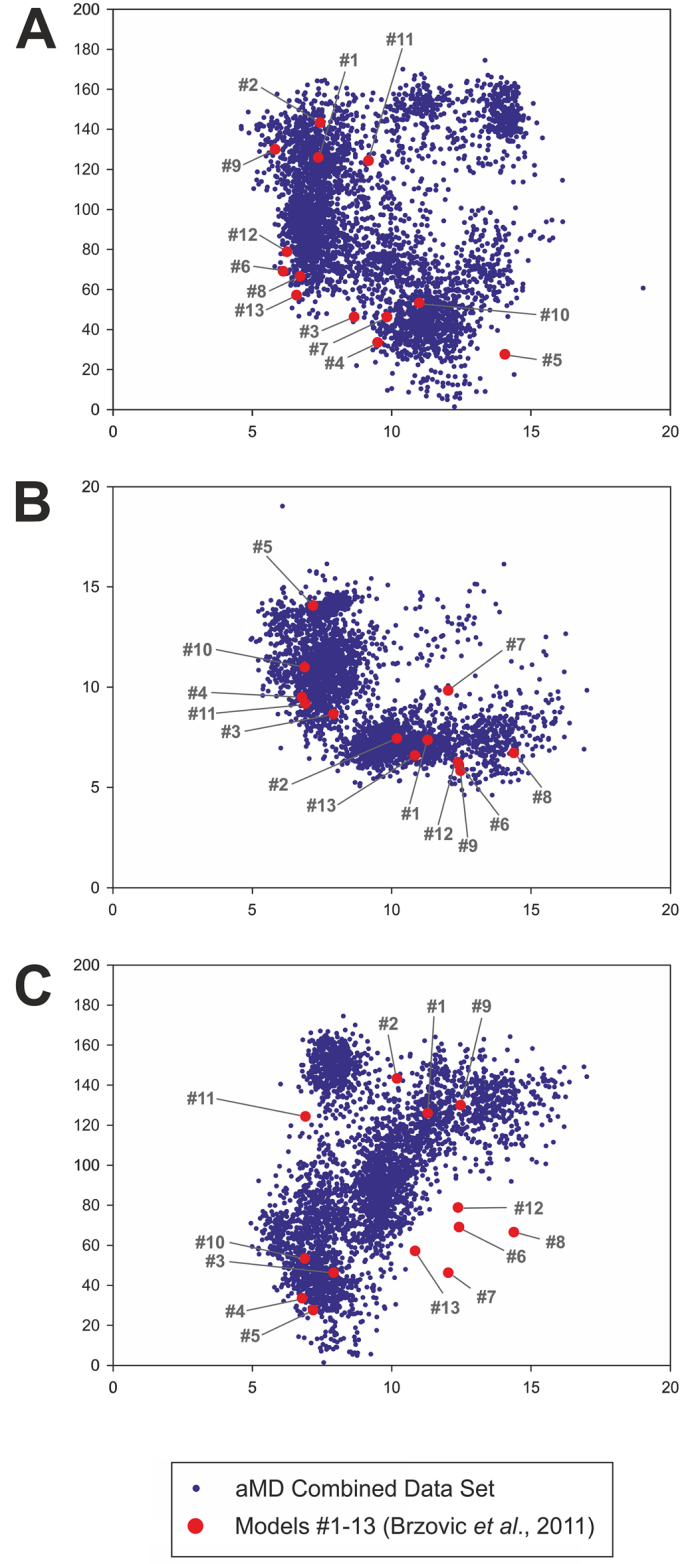
Distribution of the 13 experimentally-based models within aMD simulation phase space. Each of the blue dots defines the position in phase space of a snapshot from the four combined aMDs (GCN4-cAD/GAL11-ABD1_aMD_no 1 to 4) sampled at 1 ns intervals. The red dots define the coordinates of the 13 models calculated using identical parameters (the numbers in grey identify the model numbers as used in PDB#2LPB). The distances and angles were calculated according to the criteria described in the legends of Figs 3 and 4. **A.** Plot of the distance of F124 relative to Pocket#1 (horizontal axis; distance in Å) versus the angle of the α-helical part of GCN4-cAD relative to GAL11-Abd1 α-helix 4 (vertical axis; angle in degrees). **B.** Plot of the distance of W120 (horizontal axis) versus the distance of F124 relative to Pocket#1 (vertical axis; both distances in Å). **C.** Plot of the distance of W120 relative to Pocket#1 (horizontal axis; distance in Å) versus the angle of the α-helical part of GCN4-cAD relative to GAL11-Abd1 α-helix 4 (vertical axis; angle in degrees). There are five PDB#2LPB model structures (#6, 7, 8, 12 and 13) that are located at the periphery of the phase space simulated by the aMD simulations using this particular parameter set. These outliers are characterized by a relatively large distance of W120 from Pocket#1 (~10–15 Å) and by narrow angle (~40–80°) of the GCN4-cAD α-helical portion relative to GAL11-Abd1 α-helix 4. This set of conformations may either require more extensive sampling to be reached from 2LPB-model#1 as a starting structure, or represent conformations that are not energetically favorable under the forcefield conditions used in the simulations.

Because we started the aMD simulations from just one of the 13 different models proposed previously, we wondered to what extent he observed motions of the GCN4-cAD on GAL11-ABD1 reflected the conformational space defined by the twelve remaining models. In principle, any extensive simulation of a single member of a family of structural conformers should reveal conformations that encompass the conformations of the majority of the other family members, as these structures are expected to interconvert freely during simulation. Plots of the phase space of the combined trajectories along three coordinates (helical angle; distances of the two key hydrophobic residues (W120 and F124) relative to Pocket#1) demonstrate that approximately 87% of the model coordinates are within highly populated regions (Fig 5). We conclude that the choice of 2LPB-model#1 as the starting structure for all four aMD simulations did not result in an unusually biased sampling of conformational space.

**Fig 6:**
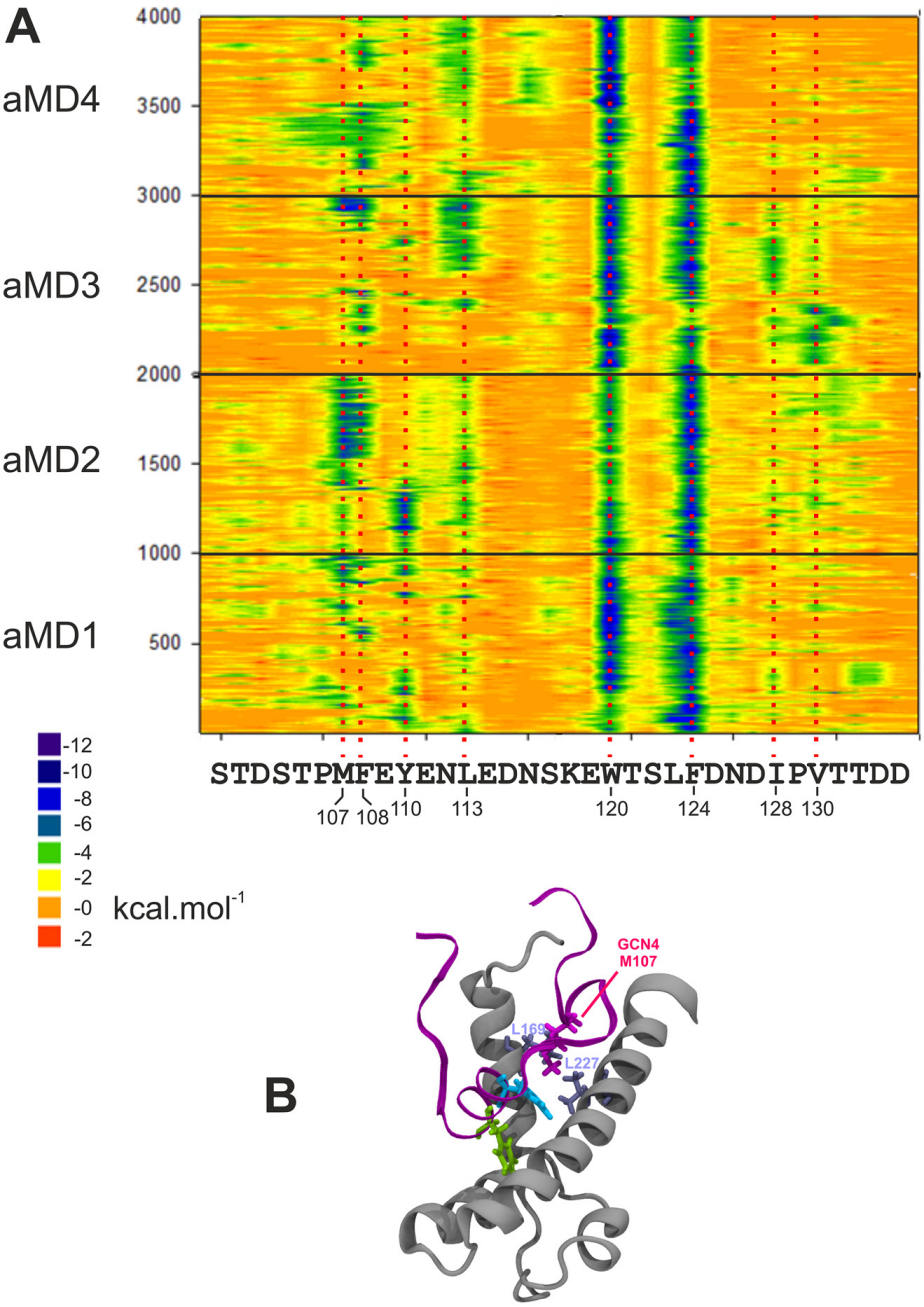
Molecular mechanics analysis of the GAL11-ABD1/GCN4-cAD interaction. **A**. The decomposition of the van der Waals contribution is shown as a contour plot. The horizontal axis represents the amino acid sequence of the GCN4 activation that was represented in the simulations and in the models in PDB#2LPB. The vertical axis represents snapshots at 1 ns intervals from the four aMD simulations. The ΔG value of the van der Waals contribution of each residue at each time point is color-coded according to the scale shown (substantial contributions to ΔG are green and dark blue). The data derived from independent simulations (indicated on the left; aMD_no1 is represented by frames 1–1000, aMD_no2 by frames 1001–2000 etc.) are shown on the same plot to facilitate the detection of constant and variable features. Residues making significant energetic contributions are highlighted by a red-dotted line aligned to the amino acid sequence. **B**. Snapshot of GAL11_GCN4_aMD_no2 (frame 935) demonstrating the hydrophobic interaction between GAL4-M107 (magenta liquorice representation) with the leucine pair GAL11-L169 and L227 (light blue liquorice representation). A movie showing the dynamics of this interaction is available (S2 Movie).

**Fig 7:**
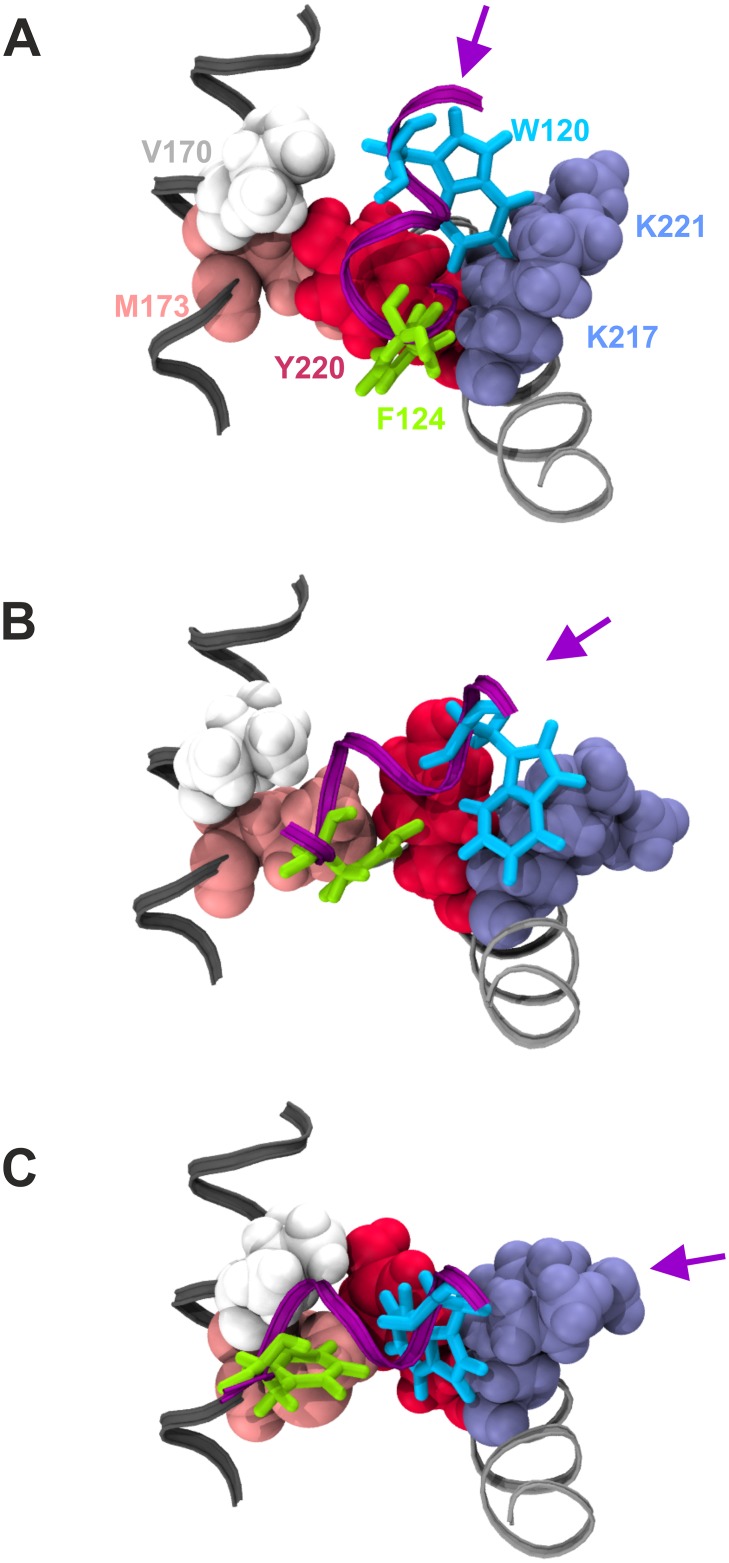
Structural basis of pocket #1 occupancy by GCN4-cAD residues W120 and F124. GCN4-cAD residues W120 and F124 are displayed in liquorice representation in light blue and green, respectively, on a portion of the GCN4 α-helix shown as a purple ribbon. Key residues participating in the formation of Pocket #1 on the GAL11-ABD1 surface are drawn as van der Waals structures and labelled according to amino acid identiy (V170 white; M173 pink; Y220 red; K217 and K 221 in light blue). V170 and M173 are part of GAL11-ABD1 α-helix 1 (see Fig 2B for helix nomenclature) marked in dark grey. K217, K221 and Y220 are on GAL11-ABD1 α-helix 4 shown in light grey. The changing orientation of the GCN4 α-helical axis is marked by a purple arrow. **A**. Structure near beginning of simulation (30 ns of GAL11-ABD1/GCN4-cAD _aMD_no1). GCN4-F124 occupies Pocket #1 formed by GAL11-Y220 and -K217 (among other residues not shown here), while GCN4-W120 engages in hydrophobic interactions with the non-polar section of the GAL11-K221 side-chain. **B**. Structure after 120 ns simulation (GAL11-ABD1/GCN4-cAD _aMD_no1). GCN4-F124 has moved towards GAL11-M173, freeing up Pocket #1 for GCN4-W120 to move in while maintaining hydrophobic engagements with GAL11-K217 and -K221. **C**. Structure after 220 ns simulation (GAL11-ABD1/GCN4-cAD _aMD_no1). GCN4-F124 binds now to GAL11-M173 and -V170, and Pocket #1 is now fully occupied by GCN4-W120.

Flexibility and structural adaptability of the cAD thus enables a highly dynamic interplay that accommodates several different combinations of pocket occupancy and helical orientation. This raises intriguing questions regarding the energetics of such a variable interaction. We calculated the molecular mechanics per-residue decomposition of free binding energy (ΔG_Binding_) measurements along the trajectories in one-nanosecond intervals using the Molecular Mechanics Generalized Bourne Surface Area (MM-GBSA) method [35]. The van der Waals decomposition data of the GCN4-cAD confirms the dominating contribution of GCN4-W120 and F124 in binding to GAL11-ABD1 (Fig 6A; electrostatic interactions play a mostly invariant role in the GAL11-ABD1/GCN4 cAD interaction: S1 Fig). Despite the major conformational changes of the GCN4 cAD relative to the coactivator surface, the energetic contributions of GCN4-W120 and F124 interactions remain relatively steady throughout all four independent simulations. A more detailed study of these interactions reveal the varying role of at least five residues within the GAL11-ABD1 Pocket #1 in mediating these contacts (Fig 7). Two hydrophobic residues, GAL11-M173 or Y220, interact with GCN4-F124 alternatively, depending on whether the F124 sidechain is located within Pocket #1 (Figs 3A and 7A), or has moved out of it and is replaced by GCN4-W120 (Figs 3C, 7B and 7C). While GCN4-F124 occupies Pocket #1, W120 makes favorable hydrophobic contacts with the sidechains of GAL11-K217 and K221 (Fig 7A–7C). The formation of alternative—but energetically equivalent—contacts thus underpins several alternative modes of binding that are conformationally quite different from each other. Another conserved residue, GAL4-L123 (Fig 2A), provides notable van der Waals contributions, mostly in conjunction with F124, and occasionally substitutes for F124 in a reversible manner (particularly obvious in simulation GAL11-ABD1/GCN4-cAD _aMD_no1; Fig 6A).

This analysis also identifies several additional residues (GCN4-M107, F108, Y110, L113, I128, and V130) as making significant additions to ΔG_Binding_, but in a distinctly non-systematic manner. The residues are nodes in a structurally highly flexible network that facilitates short-lived interactions, but do not a follow recurrent pattern due to substantial and unstable conformational changes in the GCN4-cAD. To exemplify the role of these residues, we investigated the structural interactions of GCN4-M107 in more detail. In simulation GAL11-ABD1/GCN4-cAD _aMD_no2, this residue is seen as providing a substantial van der Waals contribution lasting throughout most of the second half of the simulation (timeframe 1,400–2000 ns aMD; Fig 6A). During this time, GCN4-M107 interacts predominantly with two leucine residues, L169 and L227, which are located on two different α-helices of GAL11 (helix 1 and 4, respectively), but are spatially close to each other and interact with each other via hydrophobic interactions in the folded GAL11 structure. GCN4-M107 interacts with either residue on its own, or even with both leucines by bridging them (Fig 6B and S2 Movie).

Altogether, we interpret these findings the following way: GCN4-W120 /GCN4-F124 anchor GCN4-cAD to the GAL 11 surface, but provide no preferential stabilization of conformation and/or orientation of the cAD relative to the coactivator surface. Additional hydrophobic residues located on GCN4 contribute notable, but temporary contacts (estimated by molecular mechanics measurements to contribute only between -3 to -6 kcal.mol^-1^ each towards ΔG_Binding_). The fuzzy interaction thus results from a variable combination between two relatively strong contributors (-8 to -10 kcal.mol^-1^ each) that are regularly supported by a host of additional minor contributors subjected to continuous change. This molecular interaction pattern predicts that the binding free energy of the GCN4 cAD to GAL11-ABD1 is far from constant and subject to constant fluctuations. The MM-GBSA estimates support the idea of substantial variation in binding affinity (over a three-fold range on the micro/millisecond time scale [S2 Fig]). The half-life of the GCN4-GAL11 interaction is estimated to be in the low millisecond range [11], which supports this interpretation. Although the constant change in affinity may result in a reasonable average affinity (and may include very high affinity states), it will also drop in affinity with statistical regularity to levels that facilitate immediate dissociation. The aMD simulations, by allowing coactivator-AD domains to be monitored over longer timer frames, convey this message much clearer than the limited number of currently existing static snap-shots of models demonstrating alternative conformations [11].

### Secondary structures of ADs in presence and absence of coactivator

**Fig 8:**
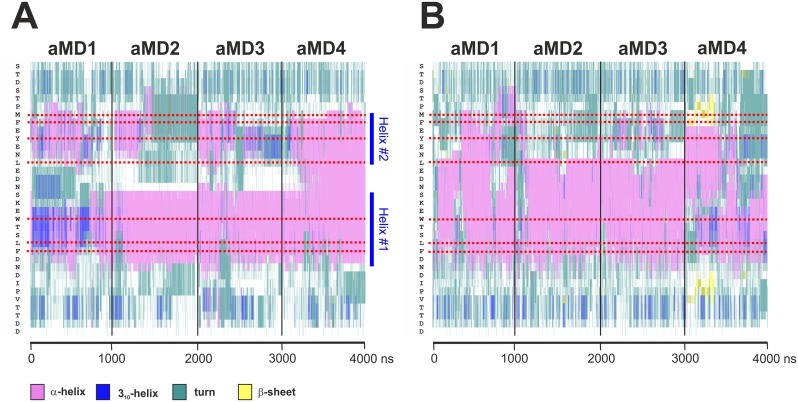
α-helicity of coactivator-bound and free GCN4-cAD. The trajectories of four aMD simulations (GCN4_aMD_no1 to no4) were combined to allow comparisons across the entire range. The secondary structure is color-coded (pink: α-helix; dark blue: 3_10_ helix; turquoise: turn; white: coil; yellow: β-sheet). The amino acid sequence is on the vertical scale (N-terminus at top; the position of key residues is marked by red dotted lines) and time in nanoseconds of aMD on the horizontal scale. **A.** Secondary structure analysis of the GCN4-cAD bound to GAL11-ABD1. Except for aMD_no1 (GCN4_aMD_no1), which shows the presence of 3_10_ helices at the beginning of the simulation, all other aMDs are almost exclusively α-helical in the region including the bulky hydrophobic residues W120, L123 and F124. There is evidence for relatively stable N- and C-terminal borders (indicated by dotted dark blue lines at residues S117 and D125, respectively), especially in aMD_no2 and 3 for the central helix ("Helix #1") that encompass W120, L123 and F124. In addition, there is evidence for the presence of an additional transient α-helix ("Helix #2") surrounding residues M107, F108, Y110 and L113. **B.** Secondary structure analysis of *de novo* folded GCN4-cAD. There is a widespread formation of short-lived α-helices at different positions and lengths that include residues W120, L123 and F124.

Having examined the molecular dynamics of the GAL11-ABD1/GCN4-cAD fuzzy complex, we turned our attention to the intrinsic structural properties of these two interaction partners. Specifically, we wanted to investigate to what extent binding of GCN4 affects the structure of GAL11 and, more importantly, how extensively the GCN4-cAD is structured on its own. We started by monitoring the formation/maintenance of secondary structure of the GAL11-ABD1/GCN4-cAD complex simulations described above. The analysis showed formation of a stable helical structure ("Helix #1") typically encompassing GCN4 residues S117 to D125 (Fig 8A). Simulation GAL11-ABD1/GCN4-cAD _aMD_no1 exceptionally shows a mixture of 3_10_-helix and α-helix during the first 750 ns of simulation before settling into a α-helical pattern, whereas all other three simulations (including the final stages of GAL11-ABD1/GCN4-cAD _aMD_no1) display extensive and stable α-helices throughout the entire time course. These results are essentially in agreement with the 13 different models presented in PDB#2LPB [11], although the simulations suggest that the C-terminal border of Helix#1 routinely extends one residue further than previously proposed to include position D125. In addition to Helix #1, the occasional presence of another N-terminally located structure ("Helix #2") is evident. Helix #2 is less stable in GAL11-ABD1/GCN4-cAD _aMD_no1, no2 and no3 and either takes up a partial 3_10_-helix conformation (GAL11-ABD1/GCN4-cAD _aMD_no1 and no3), or disappears eventually. In GAL11-ABD1/GCN4-cAD _aMD_no4, Helix #1 and #2 fuse into a single contiguous α-helix (spanning from M107 to N126 at its borders) that remains intact until the end of the simulation. Helix #2 includes the hydrophobic residues (GCN4-M107, F108, Y110, L113, I128, and V130) identified above as making occasional energetically favorable contributions to ΔG_Binding_.

We next asked to what extent the observed α-helical propensity of the GCN4-cAD was encoded within its primary structure. ADs are intrinsically disordered and are often thought to only adopt significant secondary structure upon binding to a coactivator target [11,29]. This model is, however, controversial. Whereas some NMR and circular dichroism studies of several isolated ADs claim an absence of significant secondary structure elements [11,36], other investigations suggest the presence of a significant fraction of transient α-helices [37–39] or β-sheets [40] in the unbound state of various ADs. In order to eliminate any structural "memory" from the starting structure, we constructed a model of the GCN4-cAD as a completely unfolded polypeptide from its primary amino acid sequence. After aMD simulation, any conformational changes—including the formation of secondary structure elements -will therefore solely be determined by the intrinsic properties of the polypeptide sequence itself. After a short implicit solvation minimization step to fold up the structure in a more compact random coil, we set up four independent microsecond aMD simulations under identical conditions as used previously for the aMD simulations of the GAL11-GCN4 complex (Table 1). Such simulations sample folding pathways and, especially relevant for disordered structures, reveal shifts in equilibria between short-lived conformations. The formation of α-helices occurs on the nanosecond-microsecond time scale [41] and is therefore well within the scope of the chosen simulation parameters.

An investigation of secondary structure elements formed in the GCN4-cAD aMD simulations reveals an unexpectedly high degree of spontaneously formed α-helices (Fig 8B). The formation of α-helices is especially favored in the central portion of the GCN4-cAD that contains the bulky hydrophobic residues that have been experimentally identified as critically important for the transactivation function, as well as making significant contributions to the free energy of binding to coactivators (Fig 6A). Although traces of β-sheet can be seen in GCN4_aMD_no4 (Fig 8B), these structures appear short-lived and do not support the conclusions reached by a previous study [40]. We conclude that the GCN4-cAD has intrinsic potential to form α-helical elements, even in absence of a coactivator, making it likely that these spontaneously preformed secondary structure elements represent key structural features required for coactivator interaction and binding specificity. The absence of substantial random coil elements in the region surrounding GCN4-W120, L123 and F124 allows us to postulate further that the cAD engages most likely with the coactivator with the necessary α-helices already locally preformed prior to first contact.

Although expected to have a less substantial effect, we also attempted to quantitate the effect of AD binding on the conformation of a coactivator. Consequently, we set up four independent one-microsecond aMD simulations of the GAL11-ABD1 in the absence of the GCN4-cAD (Table 1). A comparison of root mean square fluctuation (RMSF) measurements in simulations GAL11-ABD1 _aMD_no1 to no3 in the bound and unbound state shows that ABD1 becomes structurally more restricted upon cAD binding. Especially ABD1 residues involved in either pocket formation or binding of the cAD helix become less mobile (S3 Fig). GAL11-ABD1 _aMD_no4 undergoes a more substantial conformational change that includes a concerted movement of helices 1 and 2 and alters the ABD1 interaction surface. The original pocket for binding GCN4-W120 or F124 is no longer present, suggesting that this conformation of ABD1 may not be able to bind GCN4. The altered surface, however, develops new pockets, that may potentially offer alternative binding sites for other activators.

### Construction principles of a highly potent AD

Up to now, we have focused our attention on a naturally occurring AD/coactivator complex that has been shown to be physiologically relevant [11,21]. Extensive mutagenesis experiments have revealed the existence of a cryptic AD within the primary amino acid sequence of GCN4. This "cAD-like" activation domain, encompassing GCN4 residues 81–100, partially matches the structural criteria for an AD, but does not display a detectable transactivation potential (Table 2; [42]).

**Table 2:** Sequences of cAD-like motifs used in MD simulations.

GCN4_81–100_ (original sequence)	MKTVLPIPELDDAV**V**ES**FF**SS*GSGSGS*
cAD-like07	^ace^MKTVLPIPELDDAV**W**ES**LF**SS*GSGSGS*^nme^
cAD-like96	^ace^MKTVLPIPELDDA**WWWWLFW**S*GSGSGS*^nme^

The sequences correspond to the motifs tested experimentally under *in vivo* conditions [42]. The key hydrophobic residues required for AD function are shown in bold, and the linker sequence used to link the AD to the GCN4 DNA-binding domain in italics. For MD simulations, these structures were created as unfolded polypeptide chains with charge-neutralized termini (*ace* and *nme* for N- and C-termini, respectively).

Substitutions of hydrophobic residues within the cAD-like motif improve its activity and make it as potent as the GCN4-cAD. This modified cAD-like domain has proven an excellent testing ground for studying the transactivation potential of an array of directly comparable structures created by high-throughput site-directed mutagenesis [42]. We included two examples in our analyses and will refer from here onwards to the members of this collection as "cAD-like *xx*" (where *xx* stands for the transactivation potential that the sequence confers). For example, cAD-like07 refers to a 'weak' cAD-like variant that is capable of stimulating ARG3 induction ~7-fold (which is equivalent to the activation potential of the GCN4-cAD). On the other hand, cAD-like96 identifies a strong transactivator motif capable of stimulating ARG3 induction ~96-fold [42]. The system thus offers ideal conditions for further elucidation of the functional necessities of an exceptionally potent AD and its interactions with coactivators.

**Fig 9:**
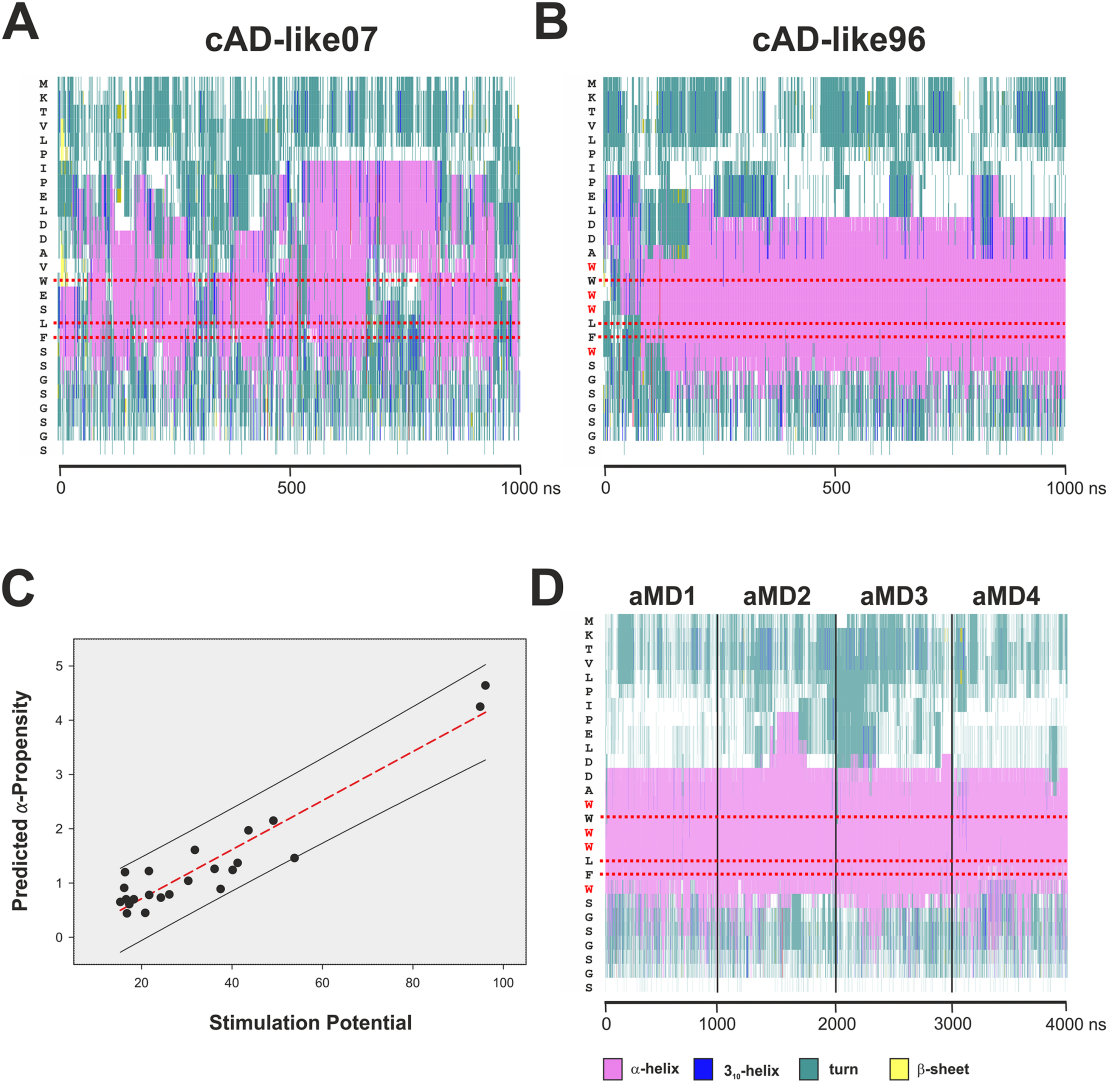
Secondary structures of cAD-like07 and cAD-like96. The complete sequence of the simulated polypeptide chain is displayed vertically on the left of each graph (Panels A, B and D). The horizontal axis represents time of aMD simulation in nanoseconds. Horizontal red dotted bars mark the positions of the conserved hydrophobic residues. A color code for secondary structure is shown beneath Panel D. **A.** Formation of α-helical structures during aMD simulation of cADlike07, displaying poor transactivation potential. **B.** Formation of α-helical structures during aMD simulation of cADlike96, with the highest observed transactivation potential. Note the more extensive formation of stable α-helices and their long-term stability in cADlike96 in comparison to cADlike07. Also, while the N-terminal boundary of the helical structures in cADlike96 is relatively sharply defined (encompassing two aspartic acid residues), the boundary in cADlike07 is much less stable and extends further N-terminal (proline residues). A comparison of the two graphs shows that there are major differences between the two cADlike domains in the extent of α-helical content and stability, as well as the position and stability of the boundaries of these helices. **C.** *Post hoc* correlation of α-helicity with transactivation potential. The α-helical propensity (vertical axis) of the cADlike sequences described in Warfield *et al*. (Table S2 in [42]) was predicted with the Agadir algorithm (http://agadir.crg.es [44]) and plotted against their corresponding transactivation potential (-fold induction of ARG3 mRNA; black dots). The red-dotted line marks the first-order linear regression (r² = 0.89) and the black lines demarcate the 95% prediction interval (including all data points). **D.** Secondary structure analysis of cAD-like96 bound to GAL11-ABD-1. The trajectories from four independent one microsecond aMD simulations (GAL11-ABD1/cAD-like96_aMD_no1 to no4; Table 1) were combined. The cAD-like96 displays stable α-helicity and helical boundaries. The conserved bulky hydrophobic residues (W94, L97, and F98; highlighted with red horizontal dotted lines) are more than 99% embedded with an α-helical context.

We set up *in silico* folding aMD simulations for the cAD-like07 and cAD-like96 motifs under identical conditions used earlier for the GCN4-cAD (Table 1). Taking into account that we previously observed significant α-helical propensity in the isolated and *de novo* folded GCN4-cAD (Fig 8B), one of the first questions we asked was whether such a propensity could also be detected in the cAD-like variants (while embedded within the same primary sequence context as used in the experimental work). The only ordered secondary structures formed under these conditions are α-helices, albeit with a noticeable difference in effectiveness. In the case of cAD-like07, contiguous α-helical regions are present fleetingly throughout most of the simulation period, but these fluctuate considerably in length and position relative to the underlying primary amino acid sequence (Fig 9A). In some instances all α-helical structures were lost, but restored in a fully reversible manner shortly afterwards. We conclude that cAD-like07 displays a notable tendency towards α-helical conformations, but these structures undergo a constant equilibrium between conformations of different α-helical content and are consequently unable to adopt a higher-order structure with a degree of stability exceeding the nanosecond range. In contrast, within the first 200 ns of aMD simulation the cAD-like96 variant adopts an extensive α-helical conformation that stably propagates afterwards and encompasses the three key hydrophobic residues (W94, L97 and F98) that mediate coactivator contact. The substitutions distinguishing cAD-like96 from cAD-like07 are four tryptophan residues (Table 2; W93, 95, 96 and 99; Fig 9B). Tryptophan is the strongest known helix conformer in short helices [43] and therefore the extensive helicity in cAD-like96 observed in the aMD simulations is in excellent agreement with expectations.

The detected differences in secondary structure content and stability between cAD-like07 and cAD-like96 strongly suggest that pronounced α-helical propensity constitutes a key factor in determining the transactivation potential of an AD, even in absence of additional conformational changes induced by binding to the coactivator surface. We tested this concept further by investigating whether there was a correlation between α-helical propensity predicted by standard bioinformatics tools and the observed effectiveness in mediating transactivation *in vivo*. A plot of predicted α-helical propensity [44] of 24 different cAD-like variants [42] against experimentally measured transcriptional simulation provides previously undocumented evidence for a strong correlation between these two variables (Fig 9C). The results show that this approach allows a direct prediction of transactivation potentials of cAD-like variants with 95% confidence using only primary amino acid sequence information.

The extensive, stable α-helicity, combined with the presence of additional bulky hydrophobic residues next and between residues W94, L97 and F98 raises some intriguing questions regarding the interaction of cAD-like96 with the GAL11-ABD1 coactivator module. As there is no structural data available for this system, we created a starting structure by *in silico* substitutions of the orthologous cAD residues in the GAL11-ABD1/GCN4-cAD NMR model (PDB#2LPB-model 1). Subsequently, four independent aMD simulations were carried out using the conditions described earlier. Just as expected from the results of the simulations of cAD-like96 on its own (Fig 9B), the cAD-like96 adopts a continuously stable α-helical conformation that includes positions W94, L97 and F98 throughout all four aMD simulations (Fig 9D).

**Fig 10:**
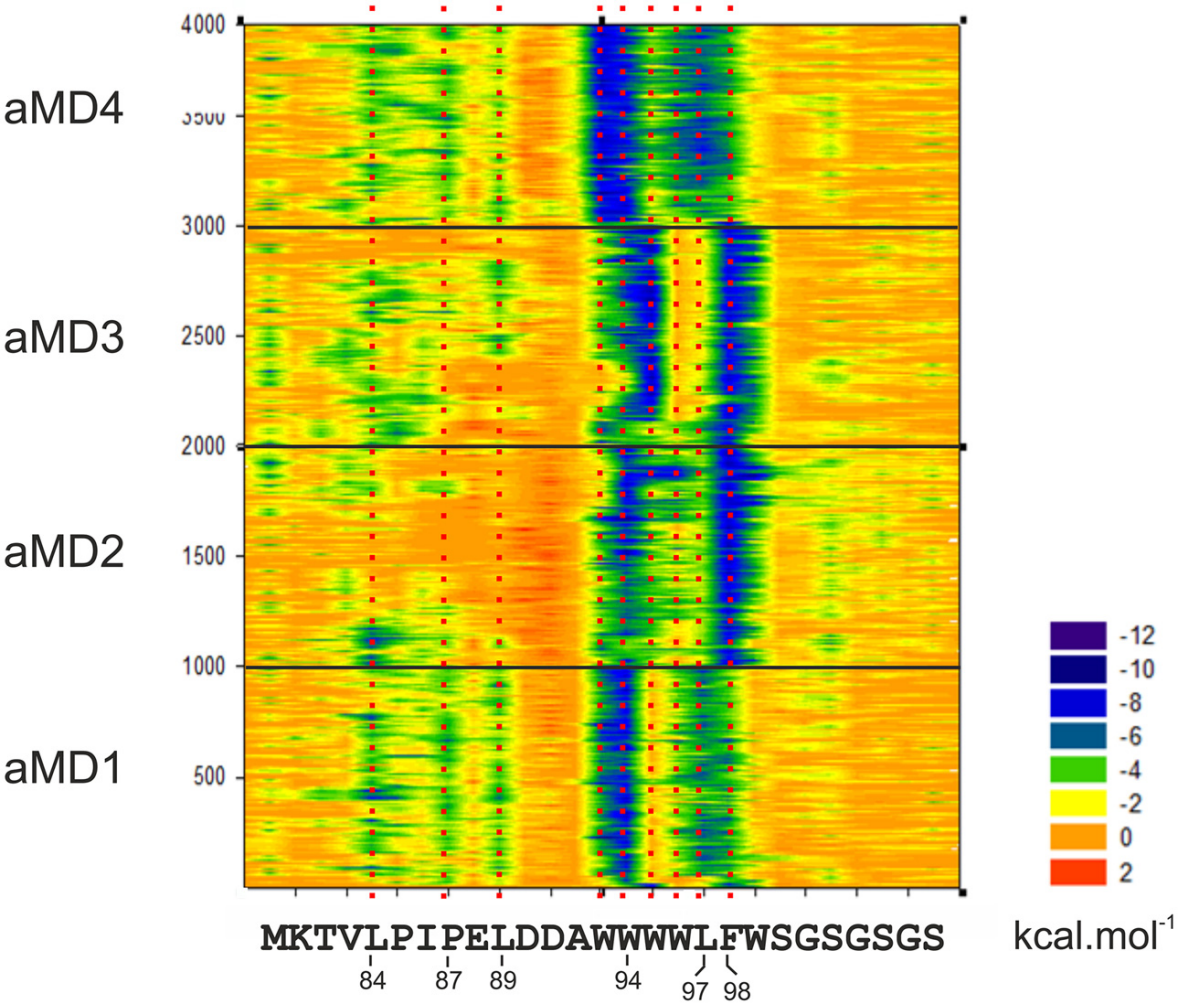
Molecular mechanics analysis of the GAL11-ABD1/cAD-like96 interaction. The decomposition of the van der Waals contribution is shown as a contour plot. The horizontal axis represents the amino acid sequence of the cAD-like96 activation. The vertical axis represents snapshots at 1 ns intervals from the four aMD simulations. The ΔG value of the van der Waals contribution of each residue at each time point is color-coded according to the scale shown (substantial contributions to ΔG are green and dark blue). The data derived from independent simulations (indicated on the left; aMD_no1 is represented by frames 1–1000, aMD_no2 by frames 1001–2000 etc.) are shown on the same plot to facilitate the detection of constant and variable features. Residues making significant energetic contributions are highlighted by a red-dotted line aligned to the amino acid sequence.

Because the cAD motif is surrounded by several additional tryptophan residues, Warfield *et al*. suggested that these tryptophans might be able to occupy the pocket in a similar manner to the original cAD motif key residues and contribute to increased binding efficiency [42]. A molecular mechanics decomposition of the van der Waals forces of the aMD trajectories of GAL11-ABD1/cAD-like96 (Fig 10) reveals interesting similarities and differences to the previously shown GAL11-ABD1/GCN4-cAD results (Fig 6A). First, the main ΔG_Binding_ contributions are once again centered on two regions (W94 and L97/F98), in addition to N-terminal contacts (L84, P87, L89) that provide fleeting contributions reminiscent of the pattern found for the GCN4-cAD (Fig 6A and 6B; note that these additional contacts, compared to GCN4-cAD [Fig 8A], are not in an α-helical conformation [Fig 9D]). It is noticeable, however, that the main contributors in cAD-like96 play a less distinct, broader role; in GAL11-ABD1/cAD-like96_aMD_no1 and no4, L97 makes a major contribution, but is distinctly supported by the flanking residues W96 and F98. Such a more diffuse energetic contribution is also observable near the W94 position. In GAL11-ABD1/cAD-like96_aMD_no3, W95 makes the dominant van der Waals contribution instead of W94 (a state that is briefly and reversibly explored in aMD2 at ~1,900 nanoseconds; Fig 10). A situation where W94 and W95 simultaneously occupy the ABD-1 pocket is not observed. As the helix would have to be distorted for these two residues to gain access to the pocket, it is unlikely for this confirmation to occur. In GAL11-ABD1/cAD-like96_aMD_no4, both W93 and W94 contribute apparently equally and create a stable configuration that remains essentially unchanged throughout one microsecond of aMD simulation conditions.

**Fig 11:**
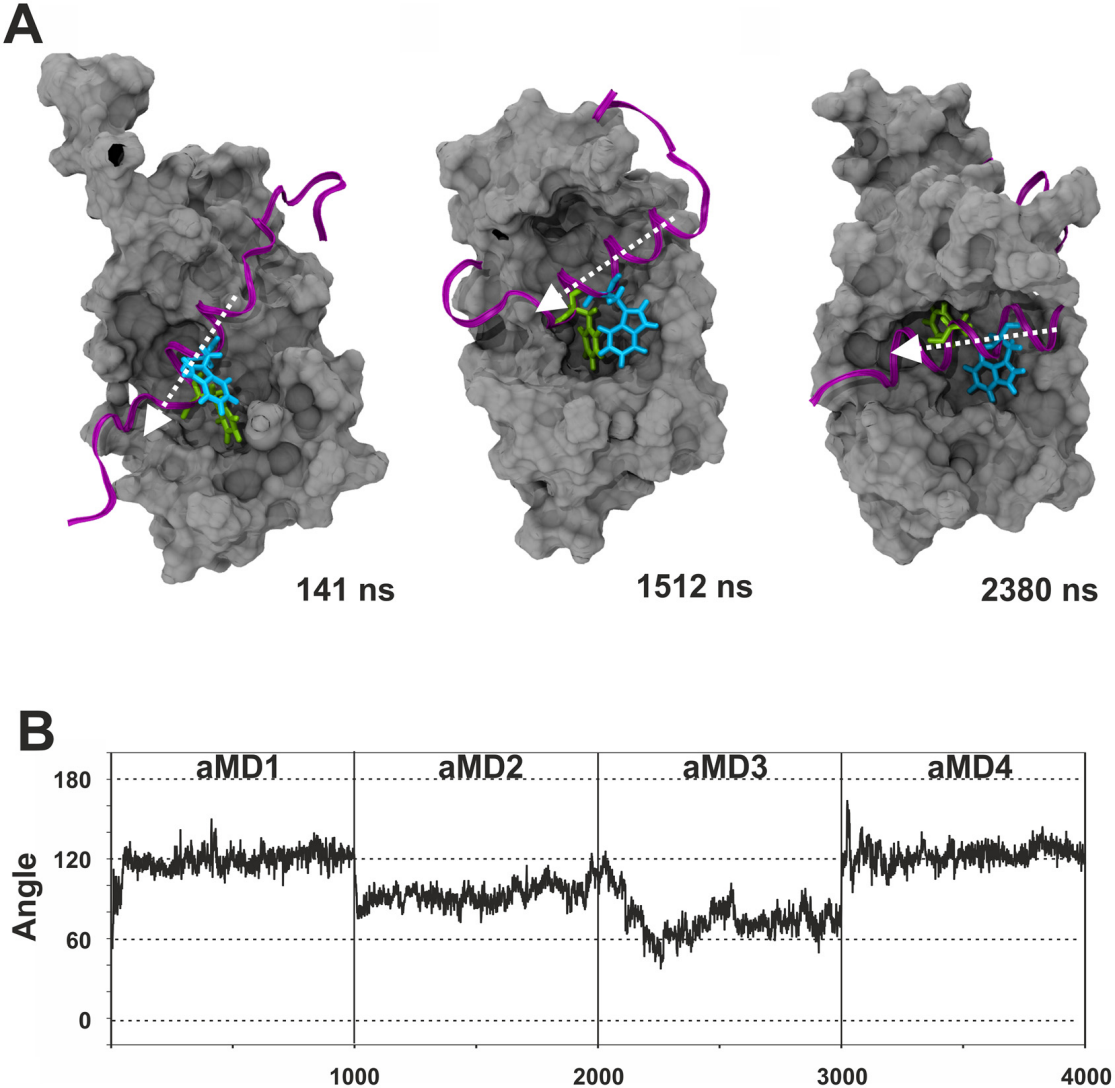
Conformational analysis of the GAL11-ABD1/cADlike96 complex. The same quantitation criteria as described in the legend for Fig 4 were applied. **A.** Examples of different helical orientations of cADlike96 relative to the GAL11-ABD1 interaction surface. **B.** Quantitation of the helical orientation.

After identification of the possible binding states, angular measurements of the helical domain of cAD-like 96 and ABD1 α-helix 4 were performed to analyse the relative orientations of these structures relative to each other. The measurements show that the main orientations observed for the cAD-like 96 helix range typically between ~60° and ~120° (Fig 11). In comparison, the GCN4-cAD helix adopts a significantly wider range of orientations (Fig 4). Consequently, even though rotations are observable for both GAL11-ABD1/GCN4-cAD and GAL11-ABD1/cAD-like96 simulations, the maximal rotation performed by the helical cAD-like 96 domain is only 60° compared to ~180° observed for the GCN4-cAD helix. The orientations also last significantly longer and do not follow the frequent and abrupt changes observed for GCN4-cAD. We conclude that overall the binding of cAD-like96 to GAL11-ABD1 is conformationally significantly more restricted and therefore reduced in "fuzziness". The increased degree of α-helicity, redundancy of hydrophobic contacts and reduced conformational freedom documented in the aMD simulations provide a quantitative base for understanding the high transactivation potential displayed by cAD-like96.

## Discussion

Synthetic Biology aims at a quantitative knowledge of molecular structure/function relationships in order to provide the tools required for reshaping the properties of living organisms in a preconceived manner. A high-level of understanding of the processes underlying gene expression mechanisms will, without doubt, be required to achieve such a goal [45]. While decades of laboratory-based investigations have identified the key players of the transcriptional machinery and revealed how they network with each other, our insights are still mostly limited to a qualitative understanding at this stage.

Molecular dynamics simulations offer the ability to model accurately the dynamic interplay of proteins with high precision. While complex systems consisting of tens- to hundreds of thousands of atoms can be studied effectively with currently available high-performance computing hardware, simulations lasting for microseconds and beyond are still challenging. Enhanced sampling methods, such as accelerated molecular dynamics (aMD) effectively extend the range by two- or three orders of magnitude into the millisecond range [33,34]. This state-of-the art technology opens the door towards a better understanding of functional interactions involving the formation of "fuzzy" complex involving intrinsically disordered molecular partners. Computational approaches are of particular relevance in an area that defies conventional experimental approaches due to the high degree of structural flexibility, the rich diversity and the short duration (half-lives typically in the millisecond range) of such interactions. Enhanced sampling method MD simulations are ideally suited for such situations and offer genuine opportunities for gaining quantitative insights into this highly relevant, but still poorly understood field.

Activation domains (ADs) are intrinsically disordered structures that have mostly defied a detailed understanding of structure/function relationships. In this study, we employed molecular dynamics simulations to model an extensively studied experimental system, the interaction between the activator GCN4 with the coactivator GAL11. The experimental identification of *in vivo* coactivator targets [21], availability of high-quality structural models [11], combined with an extensive collection of functionally characterized mutants and artificial AD variants [11,42], makes GCN4 an ideal model system for a more thorough understanding of the fundamental aspects of transcriptional activation in eukaryotic systems. The GAL11-ABD1/GCN4-cAD complex was simulated stably in multiple aMD simulations. The molecular behavior observed likely represents motions lasting hundreds of microseconds due to the acceleration parameters employed [34]. None of these simulations has yet resulted in dissociation of GCN4-cAD from GAL11-ABD1. Estimates of ΔG_Binding_ revealed, however, substantial fluctuations, that highlight the intrinsic instability of the complex and support the idea that the half-life of this complex is only in the millisecond range [11]. Longer aMD simulations under the conditions described, possibly employing reduced affinity mutants (such as GCN4-W120A or F124A; [11]), may eventually include a complete dissociation event. Similarly, long aMD simulations may reveal a real-time association of a free AD to a coactivator at a level of detail comparable to the binding of drugs to protein target sites [46].

Simulations of complexes between GAL11-ABD1 and GCN4-cAD or cADlike96 allowed us to observe a rich pattern of conformational changes that have thus far not been documented through any other experimental or theoretical approach. Using a single model as a starting structure (model 1) we confirmed essentially all aspects of the 13 different models included in the PDB structure (PDB#2LPB) and obtained an representative selection of intermediate structures (Fig 5) that can be viewed as a molecular movie (S1 Movie). The extensive collection of structures enabled us to gain insights into the constant and variable aspects of orientation of ADs relative to the GAL11-ABD1 (Figs 3, 4 and 11), changes in secondary structures (Figs 8 and 9), and key energetic contributions stabilizing the various conformers at different time points (Figs 6 and 11). Mutagenesis studies identified the cAD motif as W, L and F with spacing of i, i+3 and i+4. MM-GBSA calculations reinforced this result by demonstrating that the W and F residues are in both the cAD and cAD-like96 two major contributing residues towards binding ΔG_Binding_.

We also observed that the GCN4-cAD adopts α-helicity during aMD simulation in the complete absence of any interaction partner. The aMD simulations, starting from a polypeptide chain devoid of any secondary structure, demonstrate the formation of extensive α-helical conformations surrounding and including the conserved hydrophobic residues in a highly reproducible manner (Figs 8B, 9A and 9B). Although the presence and extent of these helices fluctuate on the hundreds of nanoseconds or microsecond time scale, the key hydrophobic residues are frequently (~65% for cAD-like07), often (~80% for GCN4-cAD) or essentially constantly (cAD-like96) arranged within an α-helical conformation, which could facilitate interactions with the coactivator surface, especially during the recruitment stage. The currently most widely—although not universally—accepted model ([37–39,47]) is based on the concept that ADs are extensively unstructured and only take up significant proportion of α-helical structures after interactions with the coactivator surface. These concepts are predominantly based on NMR-measurements showing only narrowly dispersed resonances in the ^1^H-N-dimension of various ADs, suggesting an absence of significant secondary structure elements. NMR-studies of poorly structured small domains remain, however, challenging. The energy barrier between disordered state and local α-helix conformations can be as low as 1.0–1.5 kcal/mol [48]. Differences between the intracellular environment and experimental conditions (low pH/low ionic concentrations, absence of divalent ions, effects of terminal flexibility, absence of local hydrophobic packing [49]) are likely to influence the formation and dynamics of small, unstable secondary structure elements. Apart from such experimental parameters, overlapping resonance effects in the spectra of flexible peptides, in conjunction with time-averaging phenomena can result in an underestimation of local structures in NMR experiments [50,51]. In the present case it is, however, likely that minor inaccuracies in the simulation parameters used in this study resulted in an overestimation of the stability of local α-helices. Although the Amber14SB force-field is generally not prone to overstabilize α-helical structures (in contrast to Amber ff03, CHARMM27 [52,53]), the results obtained here are in conflict with experimental data that show only 8–10% helical character in the free GCN4-cAD [11]. While the simulations correctly identify the regions displaying high α-helical propensity within the primary sequence of GCN4-cAD, it is evident that no conclusions should be drawn—as with aMD simulations in general—regarding the kinetic aspects of the data. Our conclusions regarding a recently published experimental data set based on the extensive mutagenesis of the cAD-like domain are, however, based on a different type of analysis and therefore not affected by such kinetic considerations [42]. Although not pointed out by the authors of this study, we observed a distinct correlation (r² = 0.89 for the first linear regression) between the published transactivation potential of 24 cAD-like variants and their theoretically predicted α-helicity (Fig 9C). This difference in α-helicity also emerges very clearly form the simulations of the three different ADs (GCN4-cAD, cAD-like07 and cAD-like-96) described here, both as free structures folded *de novo*, as well as complexed with GAL11-ABD1 (Figs 8 and 9). The α-helicity of a cAD-like variant can be determined using a quantitative helix-coil transition model [44], so that it should be relatively straightforward to design new cAD-like variants with predictable transactivation potentials.

The concept, that ADs either contain—or have a strong natural conformational bias towards taking up transient secondary structure elements—is not new. Experimental investigations of several other ADs has shown that they contain a significant fraction of transient α-helices in their unbound state (p53 [37]; pKID [38]; ACTR [39]; ERM [47]). We therefore conclude that eukaryotic ADs of widely different origin and specificity may contain pre-structured α-helical domains with low energy barriers of folding detectable by aMD *de novo* folding methods. Although sequence motifs with a high α-helical propensity can be clearly identified in unbound ADs in simulations, we note that there are distinct changes to the length and positions of these helices after binding to the coactivator. In the GCN4-cAD (compare Fig 8A with 8B), as well as in the cAD-like96 (compare Fig 9B and 9D), the boundaries tend to become more strictly defined (especially at the N-terminus) once bound to GAL11-ABD1, suggesting that coactivator-binding imposes a quantifiable degree of structural ordering on the AD. In addition, in the case of GCN4-cAD we detect reproducibly the formation of a previously unrecognized N-terminal secondary helix (Helix#2; Fig 8A) that spatially organizes up to four large hydrophobic residues (GCN4-M107, F108, Y110, and L113) into a structure that makes energetically significant van der Waals interactions contributions towards coactivator binding (Fig 6A and 6B and S2 Movie).

The degree of structural orderliness affects multiple key parameters [54]. A tight coupling between folding and binding may enhance equilibrium distinctions for interactions with different targets. A high degree of preformed structure may prove kinetically (dis)advantageous for binding to various target sites [55], so that the α-helical content of an unbound AD is likely to exert a significant influence on the rate of binding to various available interaction partners, even before more stable contacts are established through subsequent refolding/realigning events. The variable presence of α-helical modules (and location relative to the underlying primary sequence containing the conserved hydrophobic residues) may therefore encode a high degree of selectivity regarding to the binding of the various components of the transcriptional machinery [21].

### Outlook

The molecular mechanisms that GSTFs employ to regulate the expression of their genes are still poorly understood. There is some evidence that the binding of activation domains to basal transcription factors and coactivator complexes induces major conformational changes that could allosterically transmit signals to other components of the transcriptional machinery [56–58]. This hypothesis suggests that transient interactions of activation domains with their targets could trigger the transition between long-lived alternative coactivator conformations. Such mechanisms are, however, exceedingly difficult to study using biochemical or computational tools. In the study reported here, we have found no evidence for any significant conformational change induced in the GAL11-ABD1 structure as a direct consequence of GCN4-AD binding. Even if such changes were observed, it would still be unclear whether (and how) such an alternative conformation could be allosterically transmitted to the remainder of the GAL11 subunit (and beyond) because currently our structural knowledge of GAL11 is restricted to the ABD1 domain. An alternative—and not necessarily conflicting—view is that ADs exert most of their functions through stabilizing the assembly or position of other functional components of the transcriptional machinery, such as the basal transcriptional initiation complex. Eukaryotic promoters are potentially regulated through dozens of GSTFs bound at nearby enhancer modules, so that a multitude of energetically weak, short-lived interactions between ADs and a variety of targets could provide a significant stabilization effect through synergistic action. The short interaction half-lives and multi-target specificity of the structurally disordered ADs may under such circumstances provide the flexibility to respond to rapidly changing regulatory requirements, or provide the possibility of some components, such as RNA polymerases to "break free" of these complexes after transcription initiation. Our work documents a positive correlation between α-helicity and transactivation potential, suggesting that the overall effectiveness of AD-binding to their targets can be directly controlled through changes in α-helical propensity during evolution. Such changes may, however, have to be counterbalanced with a need for a degree of intrinsic structural disorder to sustain the ability of ADs to interact with multiple target sites. Modelling approaches, including additional AD-coactivator targets, or studying the effect of AD-interactions with larger complexes, offer great opportunities to gain further insights into the dynamical processes of coactivator-activator interactions and open numerous theoretical and applied avenues for the future. Such strategies will most likely be part of synthetic biology approaches that aim at designing artificial transcription factors with a precisely controlled range of specificity and transactivation potential in eukaryotic systems.

## Materials and Methods

### System preparation

All structures were prepared to the same specifications to maximize comparability between the simulations. For the ABD1/cAD complex simulation both polypeptide chains of Model 1 of the GCN4-GAL11 complex (PDB#2LPB) were capped (acetyl and N-methylamide groups added to the N- and C-termini, respectively) using Yasara Structure [59]. For the ABD1/cAD-like96 simulation the GCN4-cAD structure (PDB 2LPB-Model 1) was mutagenized *in silico* with Yasara Structure [59] to create the cAD-like96 sequence. The coordinates were prepared for simulation in LEaP (AmberTools 14/15) with the Amber 14SB forcefield [60], neutralized and solvated in a TIP3P [61] solvent box with a minimum distance of 15 Å between solute and border. The final ionic concentration within the water box was adjusted to a final concentration of 150 mM NaCl. Capped structures of the GCN4-cAD, GCN4 cAD-like07 and cAD-like96 were built *de novo* from their primary amino acid in LEaP, and prefolded using 10 ns of GB implicit MD before solvating them under the same conditions described above.

### Molecular dynamics simulations

The solvated models were minimized, heated to 300K and relaxed before performing a conventional MD (cMD) production run for 100 ns at a target pressure of one atmosphere to obtain values for the total potential and dihedral energy values (NPT). Simulations were carried out using the pmemd.cuda (Amber 14) applying the hybrid single/double/fixed precision model (SPFP) GPU support [62,63] using 2 fs time steps with a 10 Å cut off under control of a Langevin thermostat [64] and the SHAKE algorithm to restrain hydrogens [65]. Long-range electrostatic interactions were calculated using the Particle Mesh Ewald approximation [66]. The average total potential energies and the average dihedral energies were obtained from the cMD simulations and utilised to calculate the thresholds for dual boost aMD using an α-value of 0.2. All aMD simulations were performed with a target temperature of 300 Kelvin, and a target pressure of one atmosphere (101.325 kPa). Temperature was controlled by the Andersen temperature-coupling scheme and the pressure was controlled by the isotropic position scaling protocol applied in AMBER. Four independent 1000 ns aMD simulations were run for each structure. Details of simulations performed are summarized in Table 1. The structural models and trajectory data are available as supporting data (S1 and S2 Data Sets)

### Structure and trajectory analysis

Mapping of interaction hotspots was performed using the FTMAP algorithm (http://ftmap.bu.edu [67]). Trajectory visualisation, secondary structure analysis (based on STRIDE; [68], imaging and file conversion was performed with VMD v.1.9.2 [69]. CPPTRAJ from AmberTools 15 was utilised for distance and angle measurement [70]. Bio3D was implemented for RMSD, RMSF and principal component analysis [71,72]. Visualisation of the analytical data was performed with CRAN [73]. The MM-GBSA estimation of binding free energies was performed employing the Amber forcefield *ff99* [74] using the MMPBSA.py script [75]. Residue-specific decomposition was based on adding the 1–4 non-bonded interaction energies (1–4 EEL and 1–4 VDW) to the internal potential terms.

### α-helical propensity predictions with Agadir

The cAD sequences were acetylated at the N-terminus and amidated at the C-terminus before predicting their α-helical properties at the residue level at pH 7.0, 150 mM NaCl and 300K [44].

## Supporting Information

S1 Text Evolutionary conservation of the hydrophobic residues in the GCN4-cAD. The sequences of the central activation domain of GCN4 orthologs are shown for 28 different yeast and fungal species within the *sensu strictu* and *sensu lato* group of *Saccharomyces cerevisiae*. The near absolutely conserved residues (tryptophan [W], leucine [L] and phenylalanine [F]) are highlighted in red. Species abbreviations: *Saccharomyces cerevisiae* (*S*.*cerevisiae*); *Saccharomyces kudriavzevii* (*S*.*kudriavzevii*); *Tetrapisispora phaffii* (*T*.*phaffii*); *Vanderwaltozyma polyspora* (*V*.*polyspora*); *Kazachstania naganishii* (K.naganishii); *Lachancea thermotolerans (L*.*thermotolerans*); *Naumovozyma castellii* (*N*.*castellii*); *Naumovozyma dairenensis* (N.dairenensis); *Kloeckera africana* (*K*.*africana*); *Candida glabrata* (*C*.*glabrata*); *Kluyveromyces lactis* (*K*.*lactis*); *Kluyveromyces marxianus* (*K*.*marxianus*); *Tetrapisispora blattae* (*T*.*blattae*); *Ashbya gossypii* (A.gossypii); *Ashbya aceri* (A.aceri); *Eremothecium cymbalariae* (E.cymbalariae); *Wickerhamomyces ciferrii* (*W*.*ciferrii*); *Cyberlindnera fabianii* (*C*.*fabianii*); *Komagataella pastoris* (*K*.*pastoris*); *Zygosaccharomyces bailii* (*Z*.*bailii*); *Torulaspora delbrueckii* (*T*.*delbrueckii*); *Millerozyma farinosa* (*M*.*farinosa*); *Candida lusitaniae* (*C*.*lusitaniae*); *Meyerozyma guilliermondii* (M.guilliermondii); *Debaryomyces hansenii* (*D*.*hansenii*); *Scheffersomyces stipitis* (*S*.*stipitis*); *Candida tenuis* (C.tenuis); *Kuraishia capsulata* (*K*.*capsulata); Ogataea parapolymorpha* (*O*.*parapolymorpha*).
(PDF)

S1 Movie Differential occupation of GAL11-ABD1 pockets #1 and #2. The GAL11-ABD1 structure is shown as a surface representation in silver. The GCN4 cAD is drawn as a purple ribbon. The GCN4 key hydrophobic residues are displayed as liquorice models (W120: cyan; F124: neon green). At the start of the movie (seconds 0–8), pocket #1 is exclusively occupied by F124. Later (seconds 9–28), F124 occupies pocket #1 exclusively. Towards the end (seconds 29–32), both W120 and F124 bind to a pocket merged from the originally separate pockets #1 and #2 (see also Fig 1C). The trajectory is from GAL11-ABD1/GCN4-cAD_aMD_no1 and represents one microsecond of accelerated aMD.
(MPG)

S2 Movie Contacts between GCN4 M107 with leucines in GAL11-ABD1. The GAL11-ABD1 structure is shown as a cartoon representation in silver. The GCN4 cAD is drawn as a purple ribbon. The GCN4 hydrophobic residues W120, F124 and M107 are displayed as liquorice models in cyan, neon green and magenta, respectively. Two leucine residues in GAL11-ABD1, L169 and L227 are shown as light blue liquorice models. At the start of the movie (0–18 seconds), M107 takes up a number of different positions on the coactivator surface. M107 then establishes contact with L227 (seconds 19 to 23) and also contacting L169 (seconds 22 to 33) at irregular intervals. The trajectory is from GAL11-ABD1/GCN4-cAD_aMD_no2 and represents one microsecond of accelerated aMD.
(MPG)

S1 Fig Electrostatic aspects of the GCN4-cAD. The decomposition of the electrostatic contribution of the GCN4-cAD to binding to GAL11-ABD1 is shown as a contour plot. The horizontal axis represents the amino acid sequence of the GCN4 cAD that was represented in the simulations and in the models in PDB#2LPB. The vertical axis represents snapshots at 1 ns intervals from the four aMD simulations. The ΔG value of electrostatic contribution of each residue at each time point (calculated by MM-GBSA) is color-coded according to the scale shown. The data derived from independent simulations (indicated on the left; aMD_no1 is represented by frames 1–1000, aMD_no2 by frames 1001–2000 etc.) are shown on the same plot to facilitate the detection of constant and variable features. The contributions of most residues are essentially constant throughout the simulations.
(TIF)

S2 Fig Estimation of binding free energy of GAL11-ABD1 with GCN4-cAD. The binding free energy (ΔG) of the complex, as calculated by MM-GBSA, is shown in kcal.mol^-1^ across all four aMD simulations with data points spaced at one nanosecond aMD intervals. The boundaries between the different simulations are marked. The kcal.mol^-1^ values should be viewed as a comparative series, rather than as absolute/predicted values for the actual binding free energy because entropy effects (ΔS) are neglected. Note the large variation and irregularities in ΔG caused by the rapidly changing van der Waals contacts.
(TIF)

S3 Fig Local backbone fluctuations of GAL11-ABD1 in presence and absence of GCN4-cAD. Comparison of average root mean square fluctuations (RMSF) of the C_α_ atoms of GAL11-ABD1 _aMD_no1, no 2 and no3 unbound (blue line) and bound to GCN4-cAD (black line). Local RMSF maxima are labelled with their residue number. The diagram below shows the position of key residues involved in pocket #1 formation (red) and in contact with the GCN4-cAD Residues 120–124 (green). The mobility of the positions of GAL11 residues ~196 to ~220 becomes noticeably restricted after binding to the GCN4-cAD.
(TIF)

S1 Data Set aMD trajectories based on PDB#2LPB-model#1. The data set contains a.pdb file ('2LPB-model_no1.pdb') based on the PDB#2LPB atomic coordinates used as the starting structure for the aMD simulations. Residues numbered 1, 84, 85 and 119 are the N- and C-terminal capping residues of GAL11-ABD1 and GCN4-cAD, respectively. The GAL11-ABD1 domain is represented by residues 2 to 82, and the GCN4-cAD by residues 85 to 119. The key hydrophobic residues GCN4-W120, -L123 and -F124 are denoted by residues number 104, 107 and 108, respectively. The solvent and ion atoms present throughout the aMD simulations were removed to minimize the size of the data set. The.dcd file ('2LPB-model1_aMD_no1-4.dcd') contains 400 concatenated frames of simulation data (frames 1–100 represent 1,000 ns from GAL11-ABD1/GCN4-cAD _aMD_no1; frames 101–200 represent 1,000 ns from GAL11-ABD1/GCN4-cAD _aMD_no2; frames 201–300 represent 1,000 ns from GAL11-ABD1/GCN4-cAD _aMD_no3; frames 301–400 represent 1,000 ns from GAL11-ABD1/GCN4-cAD _aMD_no4; all frames are spaced at 10 ns intervals; Table 1). The solvent and ion atoms present throughout the aMD simulations were removed to minimize the size of the data set. The.pdb and.dcd files contain all the information required for the visualization of the trajectories using the Visual Molecular Dynamics viewer [69].
(ZIP)

S2 Data Set aMD trajectories based on PDB#2LPB-model#1, with an AD mutated *in silico* to mimic cADlike96. The data set contains a.pdb file ('ABD1-cADlike96.pdb') based on the PDB#2LPB atomic coordinates used as the starting structure for the aMD simulations. The cAD portion of was mutated *in silico* to the sequence 'cAD96' shown in Table 2. Residues numbered 1, 84, 85 and 112 are the N- and C-terminal capping residues of GAL11-ABD1 and cADlike96, respectively. The GAL11-ABD1 domain is represented by residues 2 to 82, and cADlike96 by residues 85 to 111. The key hydrophobic residues corresponding to wildtype GCN4-W120, -L123 and -F124 are denoted by residues number 99, 102 and 103, respectively. The newly mutated tryptophan residues are at position number 98, 100, 101 and 104. The alternating glycine-serine linker spans residues 106 to 111. The solvent and ion atoms present throughout the aMD simulations were removed to minimize the size of the data set. The.dcd file ('ABD1-cADlike96_aMD_no1-4.dcd') contains 400 concatenated frames of simulation data (frames 1–100 represent 1,000 ns from GAL11-ABD1/cADlike96 _aMD_no1; frames 101–200 represent 1,000 ns from GAL11-ABD1/cADlike96 _aMD_no2; frames 201–300 represent 1,000 ns from GAL11-ABD1/cADlike96 _aMD_no3; frames 301–400 represent 1,000 ns from GAL11-ABD1/cADlike96 _aMD_no4; all frames are spaced at 10 ns intervals; Table 1). The solvent and ion atoms present throughout the aMD simulations were removed to minimize the size of the data set. The.pdb and.dcd files contain all the information required for the visualization of the trajectories using the Visual Molecular Dynamics viewer [69].
(ZIP)
