# Supplementary material for: Molecular Dynamics of "Fuzzy" Transcriptional Activator-Coactivator Interactions
Source: PLoS Comput Biol. 2016 May 13;12(5):e1004935. doi: 10.1371/journal.pcbi.1004935 (PMC4866707; doi:10.1371/journal.pcbi.1004935)
Supplement: S1 Text — The sequences of the central activation domain of GCN4 orthologs are shown for 28 different yeast and fungal species within the sensu strictu and sensu lato group of Saccharomyces cerevisiae. The near absolutely conserved residues (tryptophan [W], leucine [L] and phenylalanine [F]) are highlighted in red. Species abbreviations: Saccharomyces cerevisiae (S.cerevisiae); Saccharomyces kudriavzevii (S.kudriavzevii); Tetrapisispora phaffii (T.phaffii); Vanderwaltozyma polyspora (V.polyspora); Kazachstania naganishii (K.naganishii); Lachancea thermotolerans (L.thermotolerans); Naumovozyma castellii (N.castellii); Naumovozyma dairenensis (N.dairenensis); Kloeckera africana (K.africana); Candida glabrata (C.glabrata); Kluyveromyces lactis (K.lactis); Kluyveromyces marxianus (K.marxianus); Tetrapisispora blattae (T.blattae); Ashbya gossypii (A.gossypii); Ashbya aceri (A.aceri); Eremothecium cymbalariae (E.cymbalariae); Wickerhamomyces ciferrii (W.ciferrii); Cyberlindnera fabianii (C.fabianii); Komagataella pastoris (K.pastoris); Zygosaccharomyces bailii (Z.bailii); Torulaspora delbrueckii (T.delbrueckii); Millerozyma farinosa (M.farinosa); Candida lusitaniae (C.lusitaniae); Meyerozyma guilliermondii (M.guilliermondii); Debaryomyces hansenii (D.hansenii); Scheffersomyces stipitis (S.stipitis); Candida tenuis (C.tenuis); Kuraishia capsulata (K.capsulata); Ogataea parapolymorpha (O.parapolymorpha). (PDF) [file pcbi.1004935.s001.pdf]

```
S.cerevisiae          ----------N-SKE-WTSLFDNDIPV-TTDDVSLADKAIESTEEVSLV-
S.kudriavzevii        ----------N-SKE-WTSLFDNDIPV-TTDDVSLADKAIESTEEASLV-
T.phaffii             ----------N-PKN-WTPLFDNDIAV-TAEDVNSASTAIESIADSQES-
V.polyspora           ----------N-KSSEWTSLFDNDIPV-TTDDVNSAIDAIELVEEQAIN-
K.naganishii          ----------QNPEKQWTSLFDNDIPV-VTEED-----VMFTDKAVVET-
L.thermotolerans      ----------D--PQGWNSLFDDDIAV-SVSDETIAPSVFSGSDAISEG-
N.castellii           ----------SNDPKNWTSLFENDLPIITEDDVSLNDKAIELTHDVAVN-
N.dairenensis         ----------DNDPKQWTSLFDNDIPIITEEDVVLTDKAVELTHDQVSS-
K.africana            ----------SSDPNTWTSLFDDDIPVVTEEDVTNNDDAIKITDDVAQV-
C.glabrata            ---------SSDNPENWTSLFDDDVEI-KVEDVFGADAAGASITQFEQQ-
K.lactis              -----------GGVETWTSLFDNDIPV-TLEDVSDCANAVTLELESTHNV
K.marxianus           -----------GGVETWTSLFDNDIPV-TLDDVSASANAATLELELESNA
T.blattae             KDASSSDPIANSASHEWTSLFDNDIPV-SSADVDLASKAIELIEQDPQFI
A.gossypii            -----------ADPKTWSSLFDDDIPV-TLEDVGAVEPISAAAGTE--S-
A.aceri               -----------TDPKTWSSLFDDDIPV-TLEDVSSVESITTAAGTE--S-
E.cymbalariae         -----------SNHKEWSSLFDDDIPV-TIEDVAEAQSVMTSVEVDMNT-
W.ciferrii            ------------DSENWNSLFEPNELEI--KQEETTPEPKQVSSHLIDE-
C.fabianii            ------------DSSNWTSLFEPSEMEIPVKEEKPAPIPAPVATATASA-
K.pastoris            ------------DPNNWVSLFAD--------ETTLATTPAVSRAPAASA-
Z.bailii              ------------ASGAWPAL-EPLFGE---DEERVAVEDIEEALKQVAS-
T.delbrueckii         ----------SGEESKWGSLFDDEIPINPADVFNFTPQAEEEVEVVVPS-
M.farinosa            ---------KSVSKEDWVSLFGETDGADAFAKQPFIPTDFEKSSRK----
C.lusitaniae          ---------KVNSKDDWVSLFKDEPIPEHAASAPSVPEQDEDLHNL----
M.guilliermondii      ---------KVNSSDDWVALFGDESEETSVPESSLVTHEDINLSEL----
D.hansenii            ---------KASSKDDWVSLFGGNDDSGKREETNVSMNAPTKRSY-----
S.stipitis            ---------KVNSKDDWVSLFGPAADS---DAQIISLDADIEDSLV----
C.tenuis              -----------SNKEDWVSLFDANNEIDNSFNFTKVVSDEDLSI------
K.capsulata           -----------PDADSWESLFGTEHLEPGPVEVDEKPEIVTEIKH-----
O.parapolymorpha      ------------DSSSWEPLFKEED-DLIQLAVESTPAIKEEILP-----
```

S1 Fig

Scholes & Weinzierl
