# Supplementary figures and images for: Molecular Dynamics of "Fuzzy" Transcriptional Activator-Coactivator Interactions
Source: PLoS Comput Biol. 2016 May 13;12(5):e1004935. doi: 10.1371/journal.pcbi.1004935 (PMC4866707; doi:10.1371/journal.pcbi.1004935)

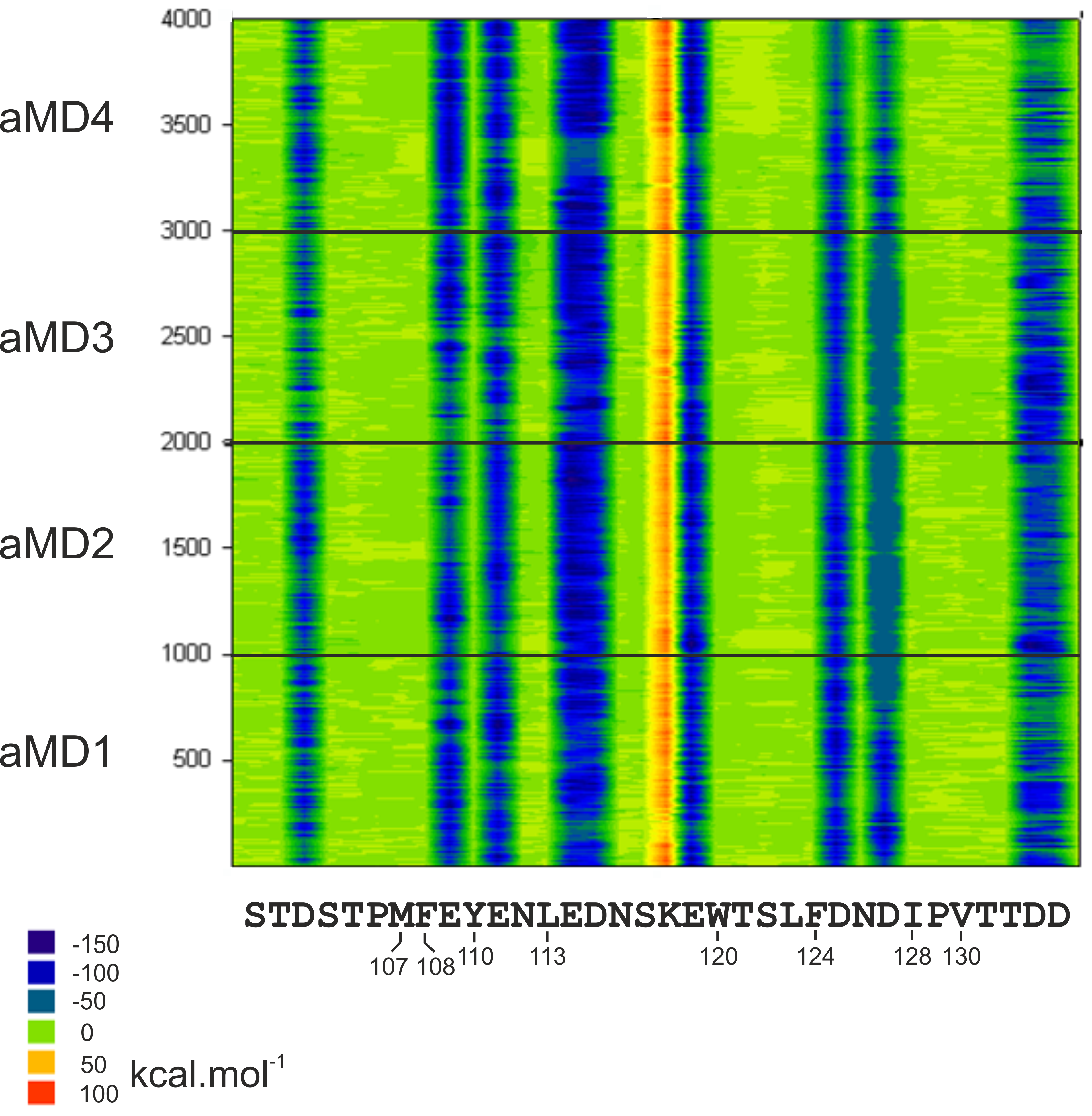

Supplement: S1 Fig — The decomposition of the electrostatic contribution of the GCN4-cAD to binding to GAL11-ABD1 is shown as a contour plot. The horizontal axis represents the amino acid sequence of the GCN4 cAD that was represented in the simulations and in the models in PDB#2LPB. The vertical axis represents snapshots at 1 ns intervals from the four aMD simulations. The ΔG value of electrostatic contribution of each residue at each time point (calculated by MM-GBSA) is color-coded according to the scale shown. The data derived from independent simulations (indicated on the left; aMD_no1 is represented by frames 1–1000, aMD_no2 by frames 1001–2000 etc.) are shown on the same plot to facilitate the detection of constant and variable features. The contributions of most residues are essentially constant throughout the simulations. (TIF) [file pcbi.1004935.s004.tif]

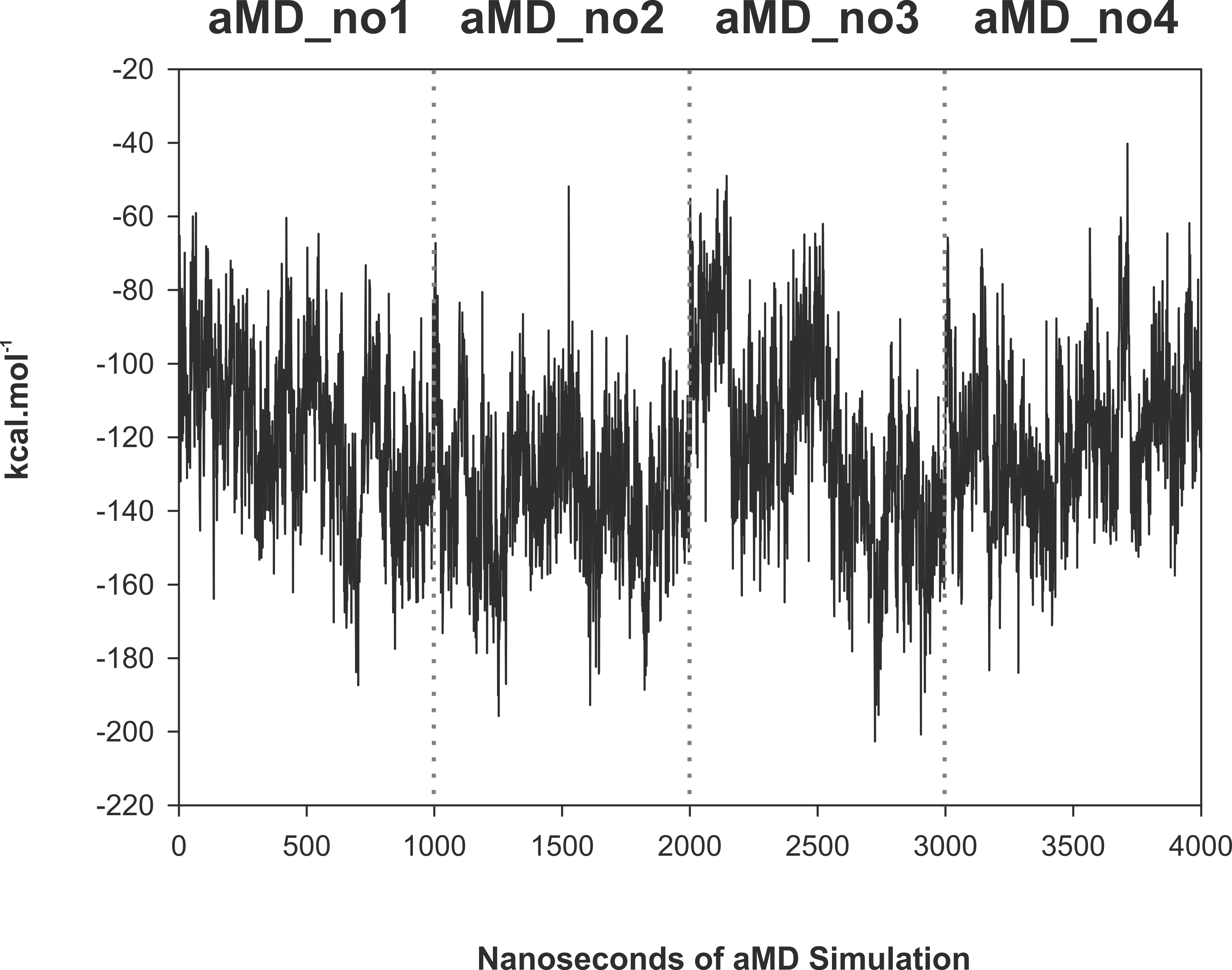

Supplement: S2 Fig — The binding free energy (ΔG) of the complex, as calculated by MM-GBSA, is shown in kcal.mol-1 across all four aMD simulations with data points spaced at one nanosecond aMD intervals. The boundaries between the different simulations are marked. The kcal.mol-1 values should be viewed as a comparative series, rather than as absolute/predicted values for the actual binding free energy because entropy effects (ΔS) are neglected. Note the large variation and irregularities in ΔG caused by the rapidly changing van der Waals contacts. (TIF) [file pcbi.1004935.s005.tif]

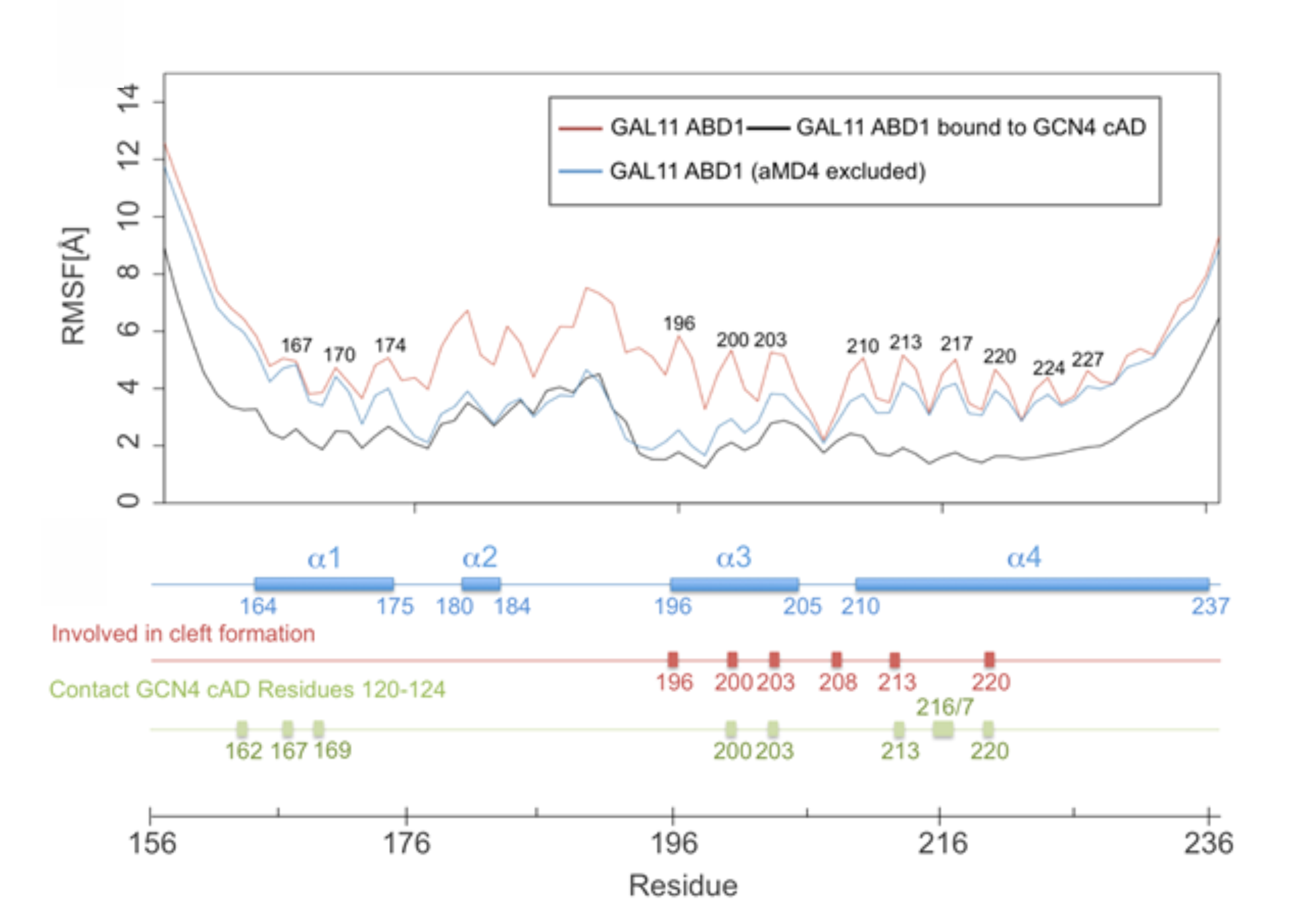

Supplement: S3 Fig — Comparison of average root mean square fluctuations (RMSF) of the Cα atoms of GAL11-ABD1 _aMD_no1, no 2 and no3 unbound (blue line) and bound to GCN4-cAD (black line). Local RMSF maxima are labelled with their residue number. The diagram below shows the position of key residues involved in pocket #1 formation (red) and in contact with the GCN4-cAD Residues 120–124 (green). The mobility of the positions of GAL11 residues ~196 to ~220 becomes noticeably restricted after binding to the GCN4-cAD. (TIF) [file pcbi.1004935.s006.tif]
